# Plant immunity to insect herbivores: mechanisms, interactions, and innovations for sustainable pest management

**DOI:** 10.3389/fpls.2025.1599450

**Published:** 2025-07-22

**Authors:** Prabhakaran Vasantha-Srinivasan, Mi Young Noh, Ki Beom Park, Tae Yoon Kim, Woo-Jin Jung, Sengottayan Senthil-Nathan, Yeon Soo Han

**Affiliations:** ^1^ Department of Applied Biology, Institute of Environmentally Friendly Agriculture (IEFA), College of Agriculture and Life Sciences, Chonnam National University, Gwangju, Republic of Korea; ^2^ Department of Forest Resources, AgriBio Institute of Climate Change Management, Chonnam National University, Gwangju, Republic of Korea; ^3^ Research & Development Center, Invirustech Co., Inc, Gwangju, Republic of Korea; ^4^ FarmInTech Co., Inc, Gokseong-gun, Republic of Korea; ^5^ Department of Agricultural Chemistry, Institute of Environmentally-Friendly Agriculture (IEFA), College of Agriculture and Life Sciences, Chonnam National University, Gwangju, Republic of Korea; ^6^ Division of Bio-pesticides and Environmental Toxicology, Sri Paramakalyani Centre for Excellence in Environmental Sciences, Manonmaniam Sundaranar University, Tirunelveli, Tamil Nadu, India

**Keywords:** plant-insect interactions, plant immunity, sustainable pest management, biotechnological approaches, climate change, plant defense

## Abstract

Plant–insect interactions pose a major threat to global food security and ecological stability. This review provides a comprehensive synthesis of the molecular and physiological mechanisms underlying plant immunity against herbivorous insects, with emphasis on structural defenses, secondary metabolites, and hormone signaling pathways including Jasmonic acid, salicylic acid, and ethylene. It highlights key advances in understanding defense signaling crosstalk, effector-triggered responses, and the role of microbiota and environmental cues. The review further discusses insect counterstrategies and explores cutting-edge technologies-CRISPR/Cas9, RNA interference, and metabolic engineering that are reshaping pest management. However, challenges remain, including limited field validation of engineered traits, ecological trade-offs, and regulatory hurdles. We conclude by outlining future research directions focused on multi-omics integration, climate-resilient defense networks, and ethical deployment of plant biotechnologies within sustainable agroecosystems.

## 1 Introduction

### 1.1 Importance of plant–insect interactions in agriculture and ecosystems

**Figure 1:**
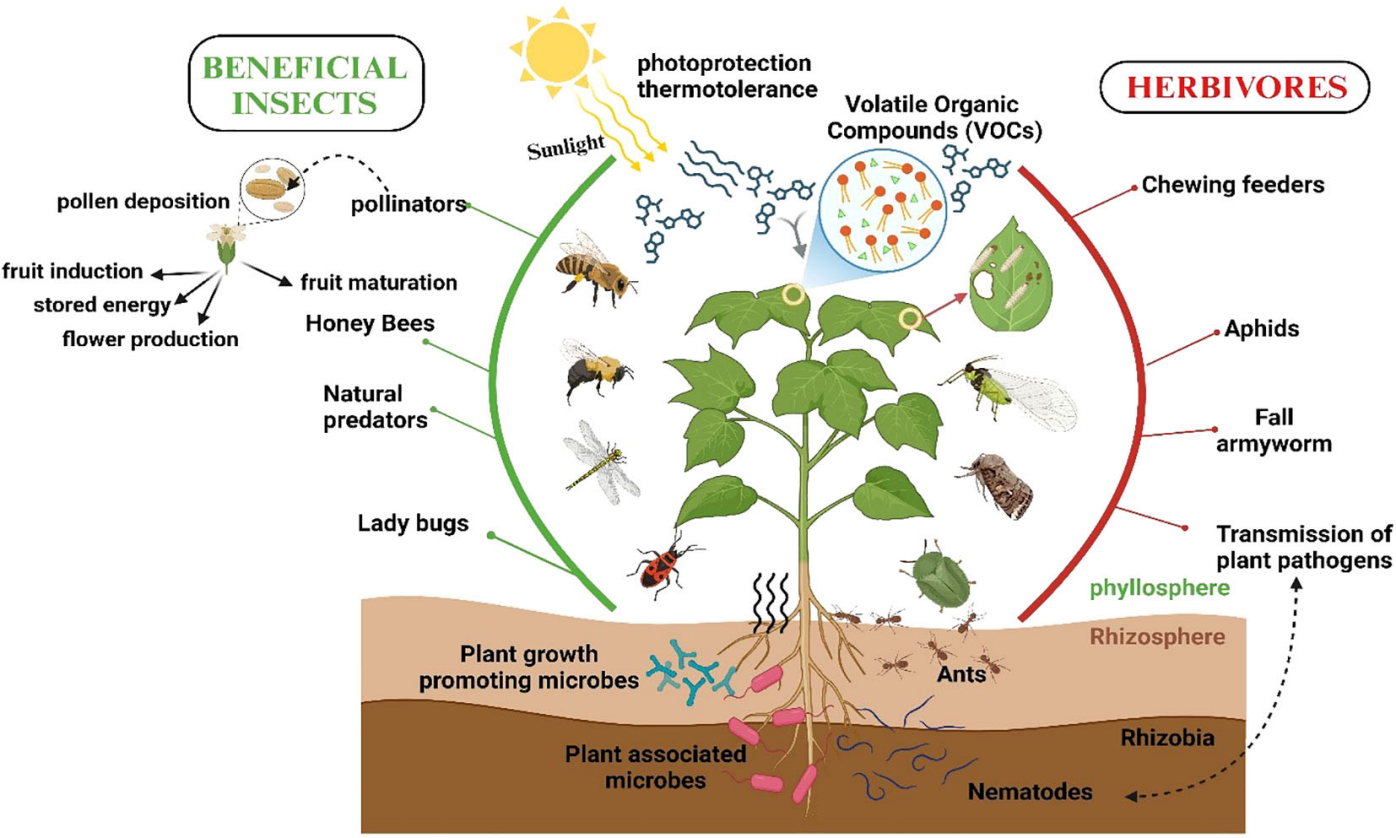
An overview of plant–insect interactions in agricultural ecosystems. Beneficial insects, such as pollinators (e.g., honey bees) and natural predators (e.g., ladybugs), support plant growth, reproduction, and defense by facilitating pollination and controlling pest populations. In contrast, herbivorous insects, such as aphids and fall armyworms, damage plants by feeding on leaves and transmitting pathogens. The rhizosphere, which consists of beneficial microbes (e.g., rhizobia), enhances nutrient uptake and plant resilience. On the other hand, some organisms, such as ants, may facilitate pest interactions, adding complexity to the ecosystem (created using BioRender.com).

Plant–insect interactions are vital to agricultural productivity and ecosystem health, influencing biodiversity, ecosystem services, and food production. These interactions can be beneficial, e.g., pollination and natural pest control, or detrimental, e.g., herbivory and pathogen transmission (Shen and Ni, 2024). In agriculture, insect pollinators, including bees and butterflies, enhance crop yields, with 75% of food crops relying on insect-mediated pollination (Riffell, 2020; Jordan et al., 2021). Predatory and parasitic insects, like lady beetles and parasitoid wasps, help regulate pests, reducing pesticide reliance and fostering sustainability (Fei et al., 2023; Wu et al., 2022). Conversely, herbivorous insects cause crop damage, impose economic losses, and spread plant pathogens (Mostafa et al., 2022; Sarwar, 2020; Wielkopolan et al., 2021). In natural ecosystems, these interactions sustain biodiversity by regulating plant populations and preventing monocultures (Balmaki et al., 2022; Whitehill et al., 2023), and coevolution between plants and insects has driven the development of traits like plant defenses and insect’s detoxification abilities (Beran and Petschenka, 2022; Endara et al., 2023; Amezian et al., 2021). Managing these interactions is key to sustainable pest management (**Figure 1**), integrating natural predators and advanced breeding or genetic approaches to reduce chemical pesticide dependence while supporting agricultural productivity and conservation (Boeraeve and Hatt, 2024).

### 1.2 Evolutionary arms race between plants and insects

The coevolution of plants and insects represents a dynamic evolutionary arms race shaping biodiversity and ecosystem functionality over millions of years (Mello and Silva-Filho, 2002). Reciprocal pressures drive plants to evolve defenses while insects develop counter-adaptations (Endara et al., 2017; Leite Dias and D’Auria, 2024). Plant defenses include physical barriers (e.g., thorns and trichomes), chemical toxins (e.g., alkaloids and terpenoids), and molecular responses like immune signaling and the production of volatile organic compound (VOC) to attract natural enemies (Salgado‐Luarte et al., 2023; Hu et al., 2024; Demis, 2024). Insects counter these defenses through detoxification systems, behavioral adaptations, and molecular effectors that suppress plant immunity (Boter and Diaz, 2023; Acevedo et al., 2015). For example, monarch butterflies exploit toxic cardenolides, using them for predator defense, while noctuid caterpillars use HARP1-like proteins to suppress plant defenses (Hoogshagen et al., 2024; Chen et al., 2019b). This coevolution drives innovation in plant immunity and insect counterstrategies, shaping both antagonistic (herbivory) and mutualistic (pollination) interactions (Bronstein et al., 2006; Nepi et al., 2018). Understanding these interactions is crucial for sustainable pest management (Dixon and Dickinson, 2024). Deciphering genetic and biochemical pathways in plant resistance and insect counter adaptation can inspire novel strategies to enhance plant immunity and disrupt insect defenses, reducing reliance on chemical pesticides and fostering agricultural resilience (Ahmad et al., 2024).

### 1.3 Benefits of research on plant immunity to insect herbivory: implications for global food security

**Figure 2:**
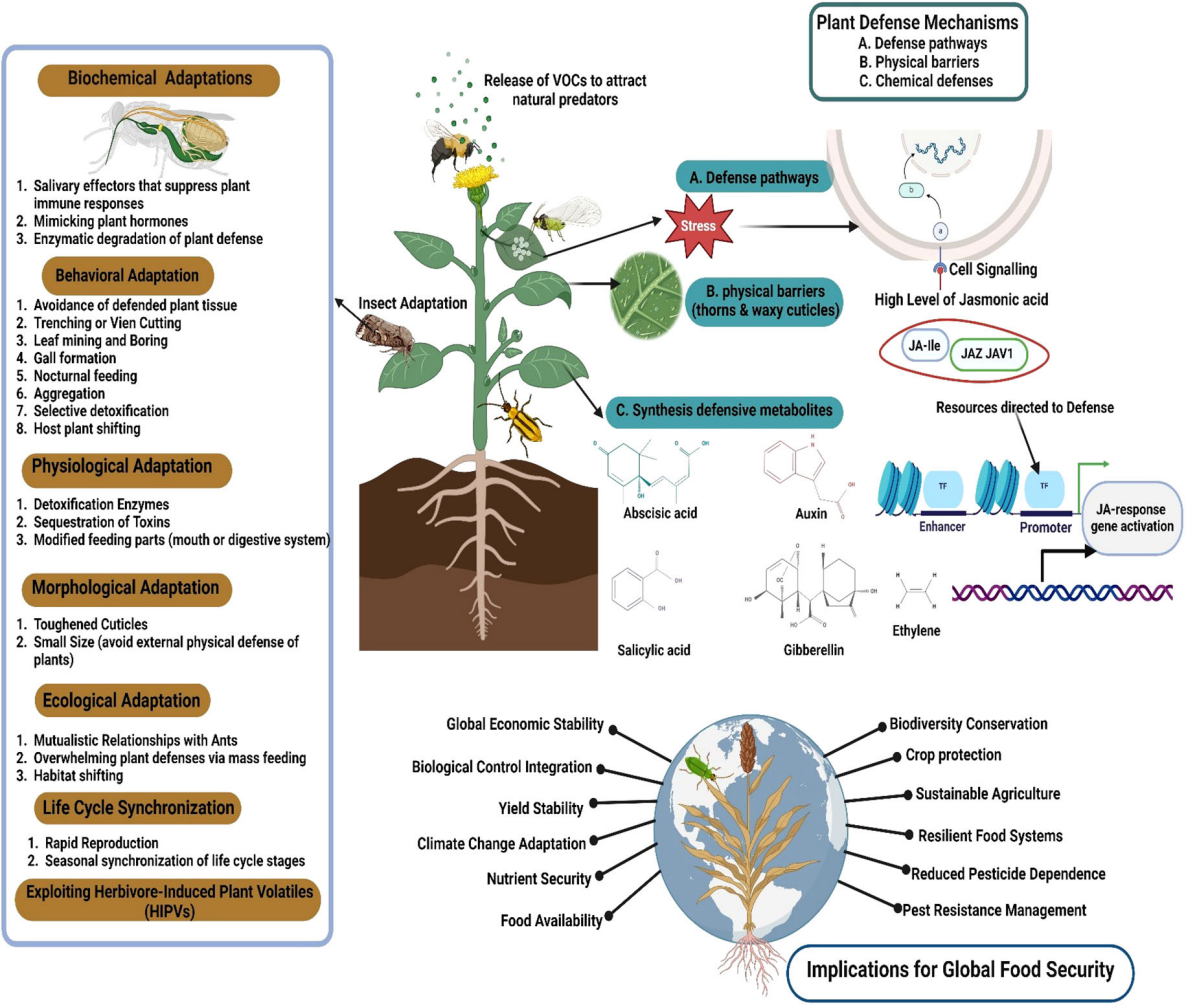
Schematic view of adaptations and defense mechanisms involved in plant–insect interactions. The figure illustrates the multifaceted biochemical, physiological, morphological, behavioral, and ecological adaptations of insect herbivores to overcome plant defense mechanisms, in addition to the implications for global food security (created using BioRender.com).

Research on plant immunity to insect herbivory is vital to addressing global food security challenges posed by climate change, pests, and diseases. Insect pests cause 20–40% of global crop losses annually, threatening food supplies and economic stability, especially in agriculture-dependent developing nations (Popp et al., 2013; Junaid and Gokce, 2024). The jasmonate signaling cascade plays a central role in mediating herbivore-induced defenses. Upon perception of damage, jasmonoyl-isoleucine (JA-Ile) accumulates and binds to the SCF^COI1 receptor complex, promoting degradation of JAZ and JAV1 repressors, thereby releasing transcription factors such as MYC2 to activate downstream defense genes, including those involved in secondary metabolite biosynthesis and protease inhibitor production (Hewedy et al., 2023; Macioszek et al., 2023; Ali et al., 2024a). This hormonal signaling cascade contributes to the synthesis of defense metabolites and structural reinforcements, such as lignin and cuticular waxes (Xiao et al., 2021; Bungala et al., 2024). Advances in breeding, genetic modification, and multi-omics integration further allow fine-tuning of these pathways for enhanced pest resilience under variable climatic conditions (Felton and Tumlinson, 2008; Soares et al., 2019; Skendžić et al., 2021). In addition, emerging studies highlight the involvement of other hormones such as abscisic acid (ABA), gibberellins (GA), and auxins in modulating plant responses to herbivory. ABA can influence stomatal regulation and drought-mediated defense trade-offs during herbivore attack. GA signaling often interacts antagonistically with JA to regulate resource allocation between growth and defense. Auxins may contribute to defense by modulating leaf morphology and influencing cross-talk with JA/SA pathways (Erb, 2018). Strengthening plant immunity reduces synthetic pesticide use, preserves beneficial insects, and fosters sustainable food systems (Sharma et al., 2021; Barbero and Maffei, 2023; da Silva Pinheiro et al., 2024) (**Figure 2**).

This review delves into the dynamic evolutionary arms races between plants and their insect herbivores, examining molecular, chemical, and physical plant defenses alongside insect counter adaptations. It emphasizes the role of environmental factors, such as climate change, in shaping these interactions. Cutting-edge biotechnological advancements, including genetic engineering and metabolic enhancement, are explored as tools to bolster plant immunity for sustainable pest management. By identifying key knowledge gaps, the review advocates for future research integrating multi-omics approaches and innovative strategies to address global agricultural and food security challenges.

## 2 Plant immune responses to insect herbivores

Plants have evolved highly sophisticated defense strategies against herbivorous insects, broadly categorized into constitutive and inducible mechanisms (Singh et al., 2024a). Constitutive defenses serve as pre-existing barriers and include structural features such as waxy cuticles, thorns, and trichomes, as well as deterrent chemical compounds like alkaloids and terpenoids, which inhibit insect feeding and interfere with their development (Malinovsky et al., 2014; Fürstenberg-Hägg et al., 2013; Balaji and Jambagi, 2024). In contrast, inducible defenses are triggered upon herbivore attack and rely on the detection of herbivore-associated molecular patterns (HAMPs) and damage-associated molecular patterns (DAMPs). These molecular cues are perceived by specific receptors that initiate intracellular signaling cascades predominantly regulated by jasmonic acid (JA) and salicylic acid (SA) pathways (Caarls et al., 2015; Ali and Baek, 2020; Snoeck et al., 2022). Additional phytohormones, including ethylene (ET) and brassinosteroids, intricately modulate these signaling networks to fine-tune the plant’s resistance depending on herbivore feeding strategy and attack severity (Jamal et al., 2013; Gilroy and Breen, 2022).

**Figure 3:**
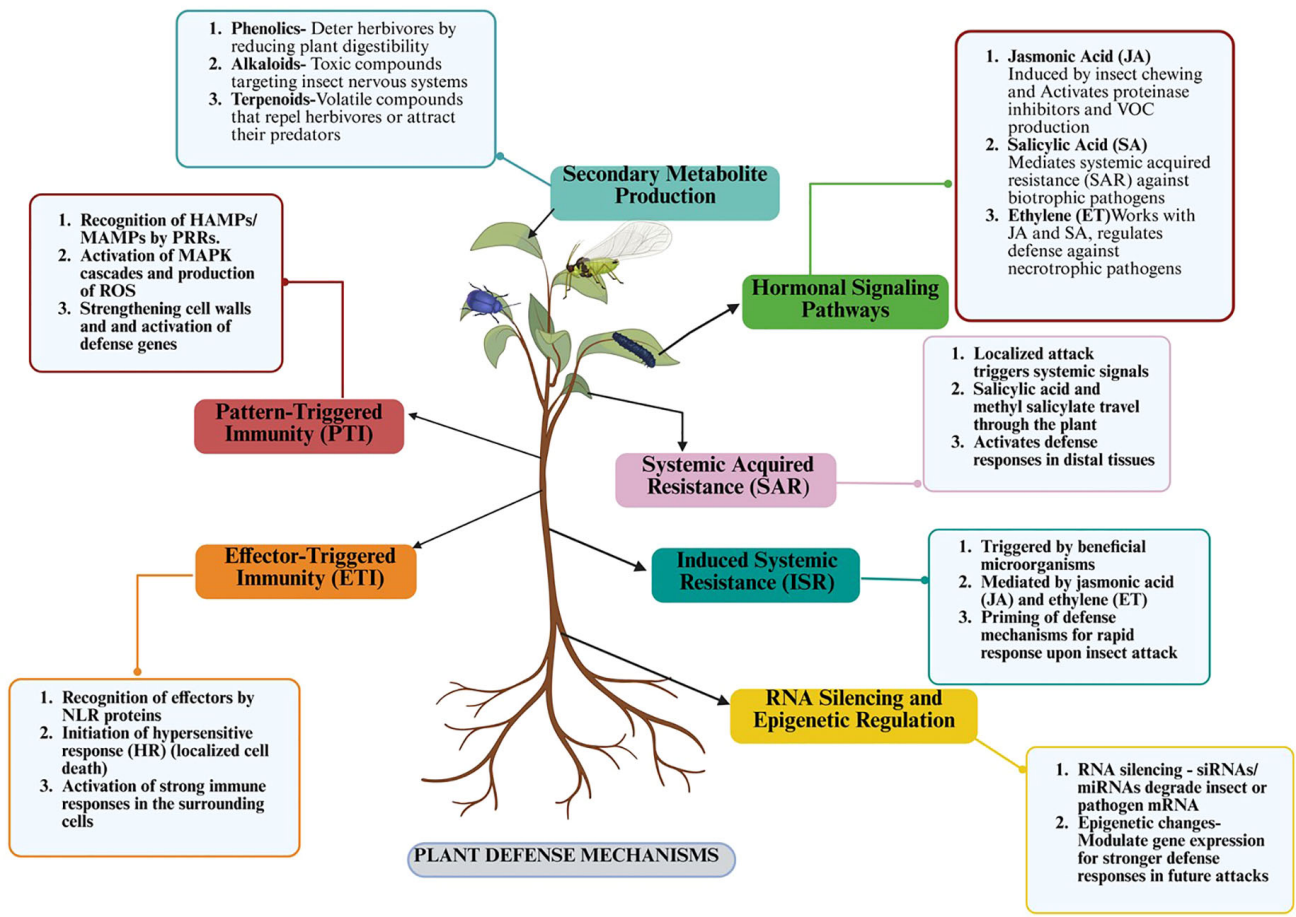
Overview of plant defense mechanisms. This figure presents the key pathways involved in plant defense against herbivores, including pattern-triggered immunity (PTI) and effector-triggered immunity (ETI), which activate defense signaling through MAPKs and NLR proteins, respectively. Hormonal pathways involving jasmonic acid (JA), salicylic acid (SA), and ethylene (ET) modulate systemic responses, such as systemic acquired resistance (SAR) and induced systemic resistance (ISR). The figure also illustrates the production of secondary metabolites and the role of RNA silencing and epigenetic regulation in enhancing plant resistance to insect attacks (created using BioRender.com).

Activation of these hormonal pathways culminates in the expression of defense-related proteins such as protease inhibitors (PIs), which disrupt insect digestive physiology by targeting gut proteases, thereby reducing herbivore growth and survival (Bezerra et al., 2021). Simultaneously, the emission of volatile organic compounds (VOCs) enhances indirect defenses by attracting natural enemies of herbivores like predators and parasitoids, thus augmenting the plant’s biocontrol potential (Bezerra et al., 2021). Beyond localized defense, systemic signaling mechanisms ensure protection of undamaged tissues via long-distance signals, including systemin, JA, and SA, which mediate systemic acquired resistance (SAR). Mobile signals such as azelaic acid further amplify systemic immunity by priming distal tissues for heightened defensive readiness (Toyota and Betsuyaku, 2022). Through this multilayered defense architecture—spanning physical, chemical, and systemic levels plants can dynamically respond to herbivore threats in varying environmental contexts (Wu et al., 2024). Deciphering these defense mechanisms is critical for developing pest-resistant crops and advancing sustainable agricultural practices (**Figure 3**).

While JA and SA signaling form the core of inducible defenses, other phytohormones such as abscisic acid (ABA), gibberellins (GAs), and auxins significantly contribute to herbivory responses, especially under concurrent abiotic stress conditions (Falconieri et al., 2022; Wang and Irving, 2011). ABA, widely recognized for its role in abiotic stress adaptation, also exerts complex influences on herbivore-induced defense pathways. Its accumulation under shade stress can inhibit bud growth, a suppression that is reversible by gibberellic acid application (Yang and Li, 2017; Bhatt et al., 2020). Moreover, ABA exhibits antagonistic interactions with JA-ET defense signaling, modulating transcriptional responses and thus affecting overall resistance (Kamle et al., 2020). For instance, ABA-mediated stomatal closure in response to herbivore attack limits water loss and preserves plant turgor pressure, indirectly contributing to stress resilience (Chen et al., 2010). Additionally, ABA can regulate secondary metabolite biosynthesis, enhancing both direct deterrence of herbivores and attraction of their natural enemies (Choudhary and Kumari, 2021).

GAs, though traditionally associated with plant growth, influence defense by modulating resource allocation between development and immunity. Depending on the context, GA signaling can either suppress or promote defense mechanisms, enabling tolerance or resistance to insect feeding. Auxins, primarily involved in cell division and elongation, have also been implicated in systemic immunity by modulating transcription of defense genes and reinforcing cell wall integrity through lignification and PR protein production (Heil, 2002). These hormones interact synergistically or antagonistically with core signaling pathways, representing an additional regulatory layer that shapes the plant’s defense landscape under biotic and abiotic stress interplay.

### 2.1 Innate immunity and pattern recognition receptors

#### 2.1.2 Pattern-triggered immunity

**Figure 4:**
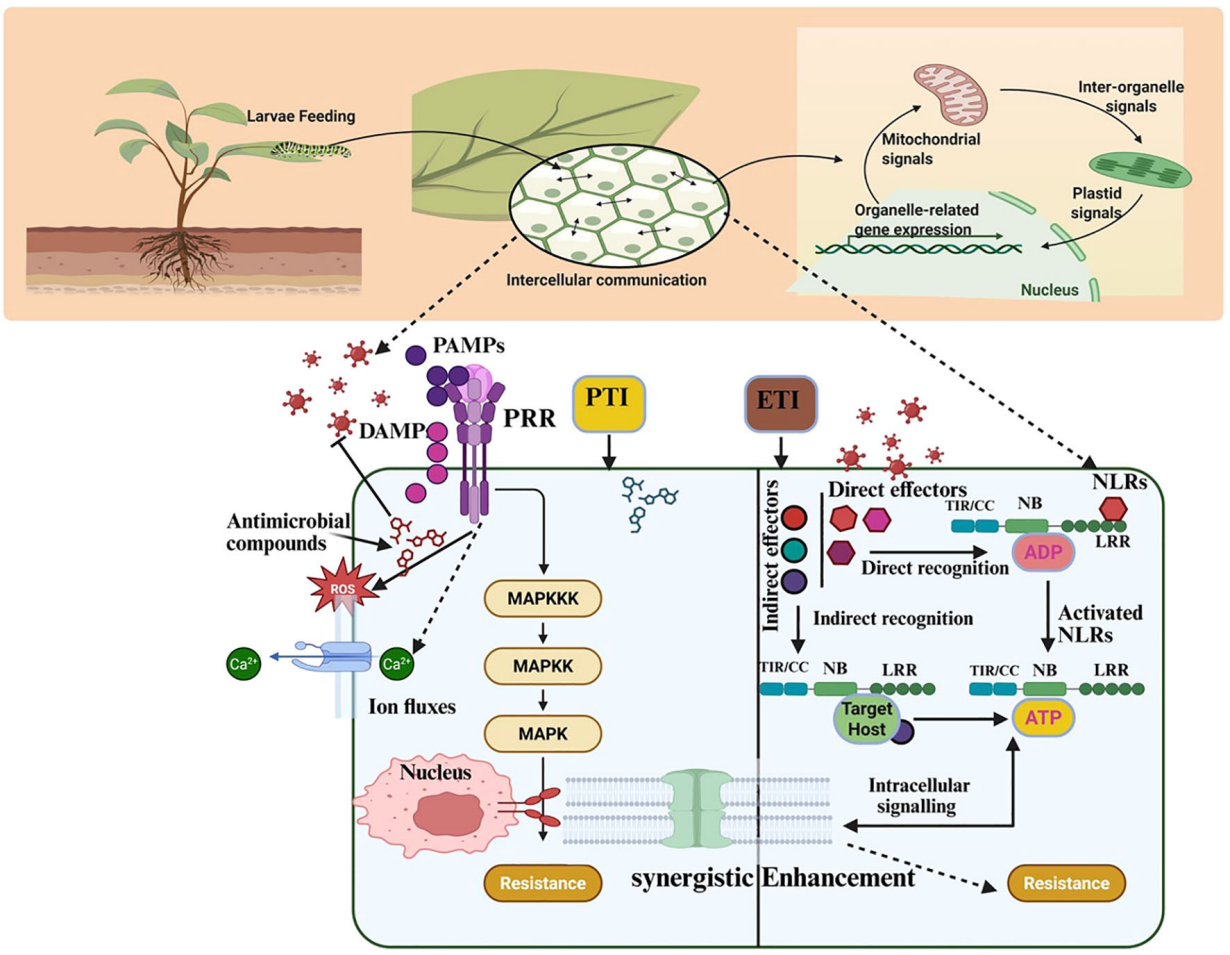
Molecular mechanisms underlying plant defense responses against herbivory. The figure illustrates the signal transduction events activated by herbivore attacks. Upon larval feeding, pattern recognition receptors (PRRs) on the plant cell surface recognize molecular patterns including pathogen-associated molecular patterns (PAMPs) and damage-associated molecular patterns (DAMPs), leading to the activation of pattern-triggered immunity (PTI). These signals activate intracellular MAPK cascades, cytosolic calcium ion fluxes, and reactive oxygen species (ROS) bursts, culminating in transcriptional reprogramming and antimicrobial compound production. Effector-triggered immunity (ETI) is also depicted, where intracellular nucleotide-binding leucine-rich repeat (NLR) proteins directly or indirectly recognize insect effectors. The synergistic interaction between PTI and ETI leads to enhanced resistance against insect herbivory. (created using BioRender.com).

**Figure 5:**
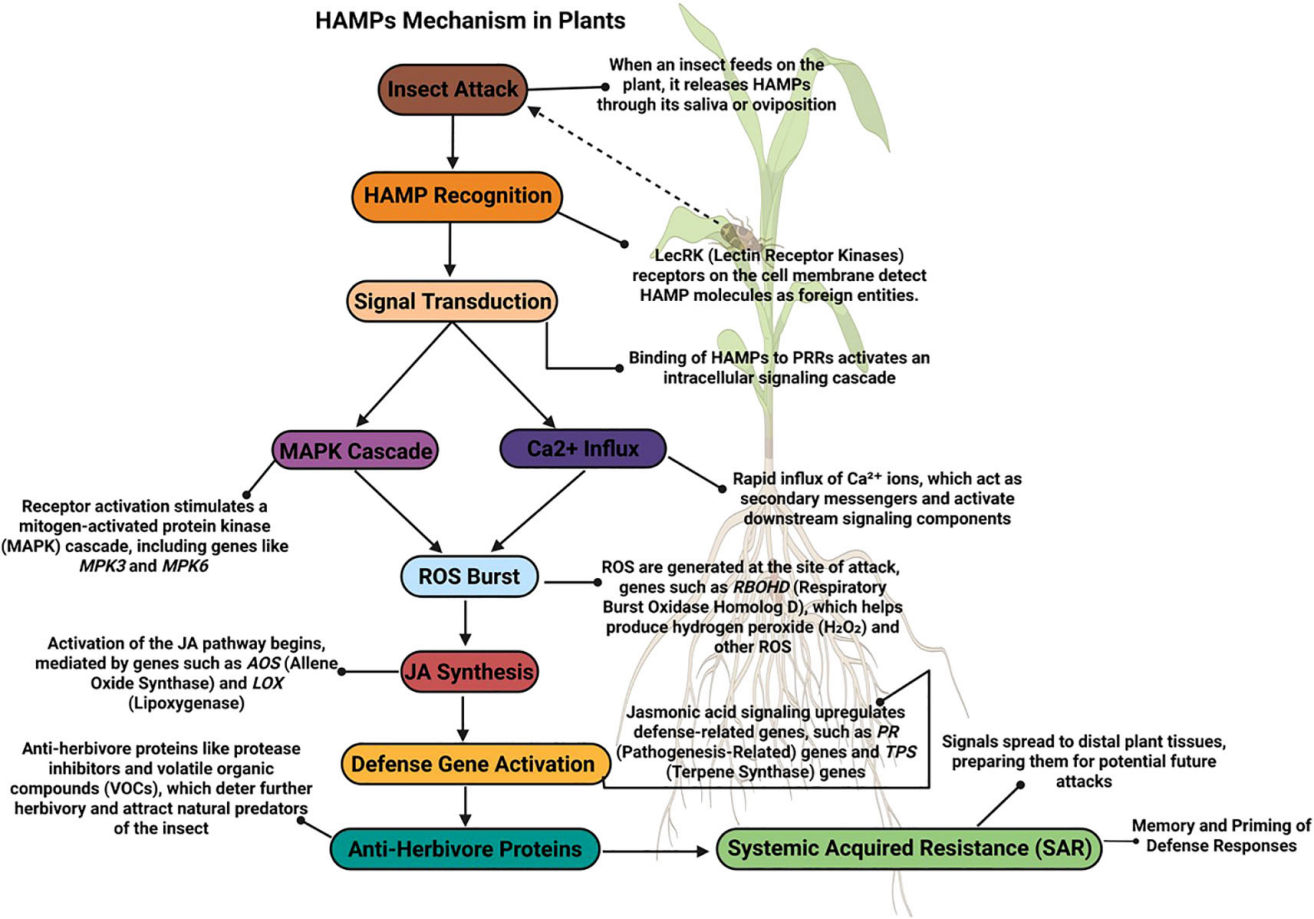
Schematic representation of HAMP pathway-mediated pattern-triggered immunity (PTI) in plants following insect attack. The diagram illustrates the sequence of cellular events in herbivore-associated molecular pattern (HAMP)-triggered responses. Insect feeding introduces HAMPs, recognized by plant pattern recognition receptors (PRRs) such as LecRKs, activating signal transduction cascades. These include mitogen-activated protein kinase (MAPK) activation and calcium ion (Ca²^+^) influx, which independently and cooperatively initiate early defense responses. Calcium influx stimulates reactive oxygen species (ROS) generation via NADPH oxidases (RBOHs), while MAPKs activate jasmonic acid (JA) biosynthetic genes such as LOX, AOS, and OPR. ROS may further amplify JA signaling and defense gene expression. This coordinated defense network results in anti-herbivore protein synthesis, secondary metabolite production, and systemic acquired resistance (SAR) (created using BioRender.com).

Plant innate immunity is a critical defense against biotic stressors, including insect herbivory. It relies on the recognition of conserved HAMPs by PRRs on plant cell surfaces, activating pattern-triggered immunity (PTI) as the first line of defense (Iriti and Faoro, 2007; Hou et al., 2019). As shown in **Figure 4**, PRRs, such as receptor-like kinases (RLKs) and receptor-like proteins (RLPs), detect HAMPs molecules from herbivore’s oral secretions, oviposition fluids, or salivary enzymes and activate intracellular signaling cascades (Singh et al., 2024b). Similarly, DAMPs, such as cell wall fragments and ATP from damaged plant cells, that signal tissue disruption trigger generalized defense responses (Harris and Mou, 2024). Together, HAMPs and DAMPs drive PTI, as illustrated in **Supplementary Figure 1**, enabling plants to target herbivores and mitigate tissue damage (Hu et al., 2024). As shown in **Figure 5**, PRRs such as RLKs and RLPs recognize HAMPs derived from herbivore saliva, oviposition fluids, or frass. In some cases, plant PRRs detect MAMPs from bacterial symbionts residing in or on herbivores. A key example is the receptor FLAGELLIN-SENSING 2 (FLS2), which binds to the conserved flg22 epitope of bacterial flagellin secreted by insect-associated microbes. Upon ligand recognition, FLS2 forms a complex with BAK1 (BRI1-ASSOCIATED RECEPTOR KINASE 1), initiating MAPK cascades, transcriptional reprogramming, and the production of defense-related compounds (Chinchilla et al., 2007; 2009; Huang and Joosten, 2024). This MAMP-triggered pathway highlights how insect herbivory may indirectly activate PTI via associated microbiota.

A key response to PRR activation in PTI is the rapid generation of reactive oxygen species (ROS), which act as signaling molecules and antimicrobial agents, causing oxidative damage to insect cells and strengthening plant cell walls (Kuźniak and Kopczewski, 2020). Concurrently, cytosolic calcium (Ca^2+^) influx activates calcium-dependent protein kinases (CDPKs), amplifying immune signaling and inducing defense-related gene expression (Gao et al., 2014; Xu and Huang, 2017), and PTI also mobilizes secondary metabolites, such as phenolics, alkaloids, and terpenoids, which deter herbivores, and PIs, which disrupt insect digestion (Gatehouse, 2011; Chowdhary and Tank, 2023). Transcription factors like WRKY (WRKY transcription factor), MYB (Myeloblastosis transcription factor), and NAC (NAM (no apical meristem), regulate these defenses, including the production of VOCs that attract herbivore predators (Pandey and Somssich, 2009; Dubos et al., 2010). Additionally, JA-mediated signaling enhances VOC production and systemic defenses (Yu et al., 2022), and crosstalk between the JA and SA pathways fine-tunes PRR-induced responses based on the type of herbivore attack, optimizing defense efficiency (Schweiger et al., 2014; Wari et al., 2022). Systemic signaling through mobile signals, like systemin, primes distal tissues for defense, boosting overall resilience (Ryan, 2000; Ryan and Moura, 2002; Delano-Frier et al., 2013).

#### 2.1.3 Effector-triggered immunity

**Figure 6:**
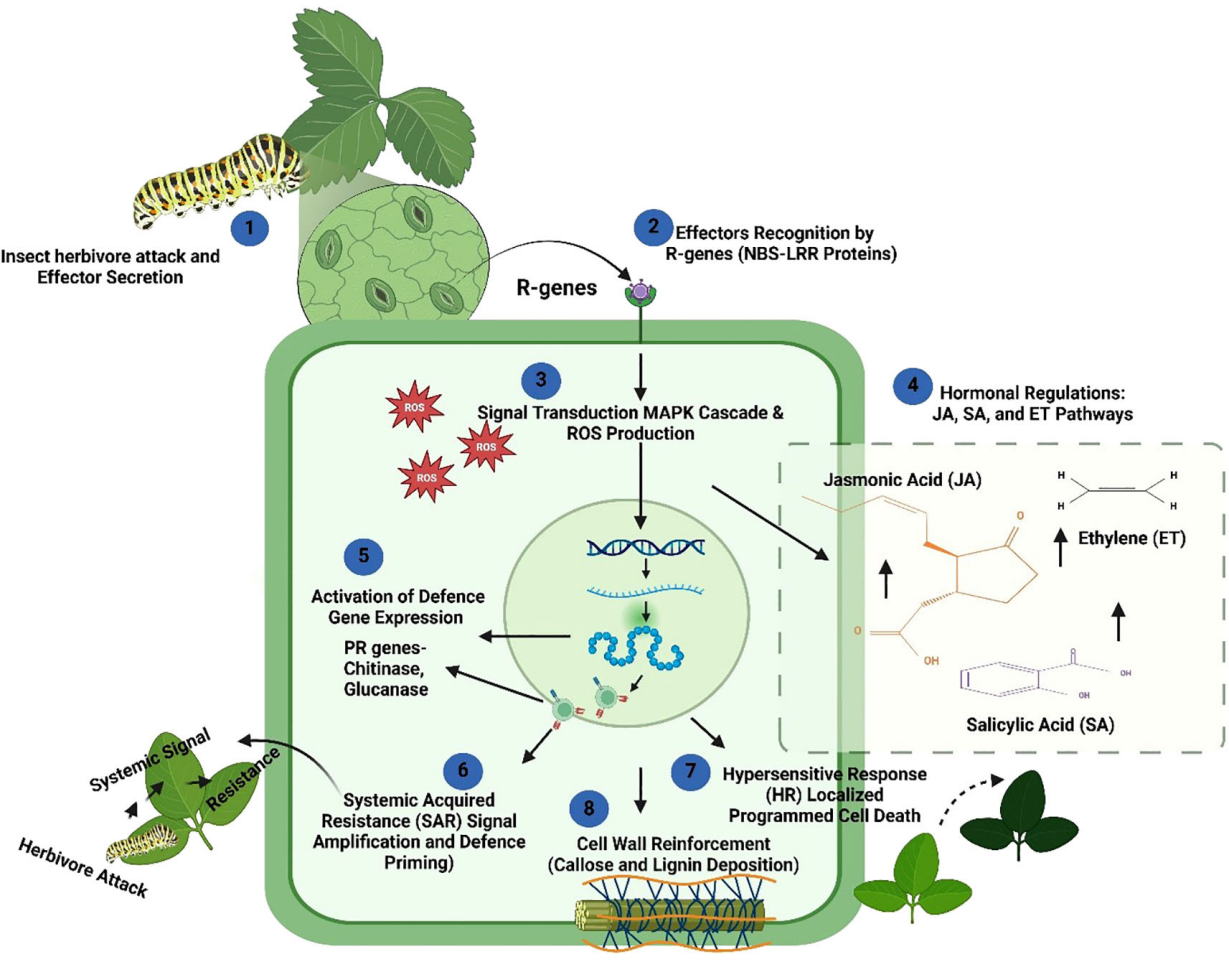
Schematic representation of effector-triggered immunity (ETI) during plant defense against insect herbivores. The ETI pathway is initiated when herbivores (e.g., caterpillars) attack the plant and secrete effectors (Step 1). R-genes, which encode nucleotide-binding site and leucine-rich repeat (NBS–LRR) proteins, recognize these insect-derived effectors and trigger the immune response (Step 2). This recognition activates a signaling cascade, including the generation of reactive oxygen species (ROS) and the mitogen-activated protein kinase (MAPK) pathway, amplifying the defense response within the cell (Step 3). Hormonal pathways, including jasmonic acid (JA), salicylic acid (SA), and ethylene (ET) pathways, are subsequently activated to further regulate immune responses. JA and ET primarily modulate responses against herbivores, while SA is more involved in SAR (Step 4). Upon signaling, defense genes, including pathogenesis-related (PR) genes, are activated and produce various proteins, such as chitinases and glucanases, to degrade the cell walls of pathogens and inhibit insect feeding (Step 5). The activation of SAR systemically propagates the immune response, priming distal tissues for potential future attacks (Step 6). The hypersensitive response (HR) is induced at the local site of attack, resulting in localized programmed cell death to limit insect feeding and pathogen spread (Step 7). Concurrently, the cell wall undergoes reinforcement through the deposition of callose and lignin, creating a physical barrier against further invasion (Step 8). Together, these molecular and cellular processes culminate in a robust defense response, curbing herbivore damage and enhancing the resilience of plants against insect pests (created using BioRender.com).

Effector-triggered immunity (ETI) is a specific plant defense mechanism activated by pathogen- or insect-derived effectors, complementing PTI as a second layer of immunity (Tsuda and Katagiri, 2010). This specialized system enables plants to counter herbivore attacks, making them crucial to agricultural productivity and ecological stability (Nguyen et al., 2021). ETI relies on resistance genes (R-genes) encoding nucleotide-binding site (NBS) and leucine-rich repeat (LRR) proteins (R-proteins collectively), which detect insect effectors directly or indirectly via guard or decoy models (Van der Hoorn and Kamoun, 2008; Wu et al., 2014). Upon effector recognition, these R-proteins activate defense cascades that enhance resistance (Kaur et al., 2021), and recent studies suggest that R-genes have broad-spectrum potential, targeting both pathogens and herbivores (Zhang et al., 2022; Wang et al., 2023). The ‘guard hypothesis’ posits R proteins monitor specific host proteins termed ‘guardees’ which are common targets of pathogen effectors. When these guardees are modified by effectors, the R proteins detect these changes and trigger effector-triggered immunity (ETI) to counteract the pathogen attack (Van der Biezen and Jones, 1998; Dangl and Jones, 2001). In the “decoy model,” plants evolve decoy proteins resembling herbivore targets to bait effectors, ensuring precise detection and response (Wang et al., 2021a). This dynamic recognition system allows plants to counter biochemical manipulations by herbivores and tailor molecular defenses (**Figure 6**).

Upon recognition, R-proteins trigger ROS accumulation, MAPK cascades, and defense gene expression, leading to localized programmed cell death, which limits insect damage (Gogoi et al., 2024; Zhang and Zhang, 2022). Overall, ETI is tightly regulated to balance defense strength with cellular homeostasis (Falak et al., 2021). Unlike the broad-spectrum resistance of PTI, ETI is highly specific, targeting unique insect-derived effectors (Zhang et al., 2024a). For example, *Nicotiana* species possess R-genes conferring resistance against *Helicoverpa armigera*, while *Arabidopsis thaliana* has R-genes targeting *Pieris rapae* (Chen et al., 2022; De Vos et al., 2006). This specificity ensures efficient resource use and effective defense. Additionally, R-genes contribute through antimicrobial activity, structural barrier enhancement, and immune signaling amplification (Farvardin et al., 2024). Advances in high-throughput sequencing and CRISPR (Clustered Regularly Interspaced Short Palindromic Repeats) technology have identified novel R-genes and enabled transgenic approaches to enhance pest resistance in crops (Tailor and Bhatla, 2024). Strategies like gene pyramiding, stacking multiple R-genes, and synthetic biology approaches engineering R-proteins with improved specificity offer promising solutions to combat insect adaptation and resistance (Das et al., 2022; Vo et al., 2023).

### 2.2 Plant signaling pathways involved in defense

Plants deploy highly coordinated signaling pathways to mount rapid defense responses against herbivores, wherein jasmonic acid (JA), salicylic acid (SA), and ethylene (ET) function as primary regulators (Romero et al., 2023). Upon herbivory, signaling cascades are activated almost immediately after damage through wounding perception and herbivore-associated molecular pattern (HAMP) recognition, leading to early hormone production within minutes (Machado et al., 2016; Pandey et al., 2017; Zafeiriou et al., 2022). These hormonal networks regulate both direct defenses, such as protease inhibitors (PIs), oxidative enzymes, and secondary metabolites that impair herbivore digestion, and indirect defenses including herbivore-induced plant volatiles (HIPVs) that recruit natural predators (Sultana et al., 2024; Upadhyay et al., 2024). Recent studies have highlighted that indole-3-acetic acid (IAA) plays a pivotal role in the early systemic signaling following herbivore attack, especially during insect wounding (Singh et al., 2024a). IAA accumulation is often triggered within minutes after herbivore perception, preceding the JA burst, and coordinates auxin-responsive gene expression that modulates downstream defense amplification and tissue remodeling (Machado et al., 2016; Ali et al., 2024c).

#### 2.2.1 JA: the principal hormone involved in defense against herbivory

JA biosynthesis is initiated almost immediately after herbivore damage, often within minutes, as demonstrated in multiple species including *Arabidopsis*, chickpea, and *Nicotiana* (Machado et al., 2016; Pandey et al., 2017; Zafeiriou et al., 2022). Tissue damage activates the octadecanoid pathway, converting α-linolenic acid into jasmonoyl-L-isoleucine (JA-Ile), which interacts with the SCF^COI1-JAZ complex to release MYC transcription factors that regulate downstream defense genes (Macioszek et al., 2023; Hewedy et al., 2023; Ali et al., 2024a). Within minutes, this signaling cascade induces the production of direct defense compounds, including alkaloids, terpenoids, and PIs that impair insect digestion (War et al., 2018; Kumar et al., 2024). Concurrently, JA regulates oxidative defenses through polyphenol oxidases (PPOs) and ROS generation that inflict further tissue damage on herbivores (Taranto et al., 2017). JA also activates HIPVs that attract predators and parasitoids, contributing to indirect defense strategies (Paudel Timilsena et al., 2020).

#### 2.2.2 SA: modulator of crosstalk and indirect defense

Although primarily associated with pathogen defense, SA also modulates responses to herbivores, particularly phloem-feeding insects, through rapid activation of SA biosynthesis pathways following localized cell damage (Pandey et al., 2017; Hou and Tsuda, 2022). Piercing-sucking herbivores like aphid’s trigger SA signaling via the isochorismate pathway, where Isochorismate Synthase 1 (ICS1) mediates SA biosynthesis in chloroplasts (Arif et al., 2021). SA activates NPR1-mediated transcription of defense-related genes including PR genes (Backer et al., 2019; Christopher et al., 2003). Crosstalk between SA and JA is largely antagonistic, allowing fine-tuned regulation based on herbivore feeding strategy (Yang et al., 2015), though synergistic cooperation may occur during combined pathogen-herbivore challenges (Mishra et al., 2024a). Additionally, SA regulates volatile and nectar production, indirectly influencing herbivore control via recruitment of natural predators and pollinators (Al-Khayri et al., 2023).

#### 2.2.3 ET: enhancer of herbivore defense responses and synergist of JA signaling

Ethylene operates synergistically with JA, often enhancing defense responses especially during extensive tissue damage (Pandey et al., 2017; Zafeiriou et al., 2022). ET biosynthesis is rapidly induced following herbivory, starting with methionine conversion to 1-aminocyclopropane-1-carboxylic acid (ACC) by ACS and subsequent oxidation to ET by ACO enzymes. ET perception via ETR1 and downstream signaling through EIN2 and EIN3/EIL transcription factors amplifies JA-driven responses, upregulating genes involved in PIs, PPOs, and ROS production (Khan et al., 2024; Bungala et al., 2024). ET also promotes cell wall reinforcement through lignin biosynthesis and callose deposition, limiting further herbivore penetration (Wang et al., 2020; Ninkuu et al., 2022; Xiao et al., 2021; Shi et al., 2016). The synergistic regulation of PDF1.2 by JA-ET pathways provides defense against necrotrophic herbivores (Koornneef and Pieterse, 2008).

#### 2.2.4 ABA: coordinator of defense under abiotic-biotic stress intersection

The co-occurrence of drought and herbivory imposes multifaceted stress on plants, necessitating a hormonal crosstalk to orchestrate defense and survival. Abscisic acid (ABA), classically known for regulating abiotic stress responses, plays a critical role in modulating herbivore-induced defenses, especially under drought (Mundim and Pringle, 2018). ABA accumulation mediates stomatal closure, osmotic balance, and root growth by activating stress-responsive genes such as RD29A and NCED3 (Zhang et al., 2023b). Under simultaneous drought and insect attack, ABA interacts with JA and ET pathways to fine-tune defense priorities (Tabaeizadeh, 1998; Aslam et al., 2022). For instance, ABA-mediated stomatal closure reduces transpiration but also limits volatile emission, thereby modulating herbivore recognition and natural enemy attraction (Liu et al., 2022; Cardoso et al., 2020). Additionally, ABA influences the synthesis of defensive secondary metabolites and stress-induced proteins, contributing to both direct and indirect defenses (Pri-Tal et al., 2023). Herbivore stress can also suppress photosynthesis by downregulating the 2-C-methyl-D-erythritol-4-phosphate (MEP) pathway, limiting isoprenoid-derived defenses (Mitra et al., 2021). Importantly, ABA signaling is interconnected with SA pathways, forming a regulatory hub in drought-herbivory resistance (Benderradji et al., 2021). Beyond defense, ABA orchestrates developmental adjustments such as seed dormancy and root-shoot architecture to optimize survival under compounded stress (González‐Guzmán et al., 2014; Wang et al., 2018; Huang et al., 2018; Felemban et al., 2019).

#### 2.2.5 Crosstalk and integration of JA, SA, and ET in defense against herbivores

**Figure 7:**
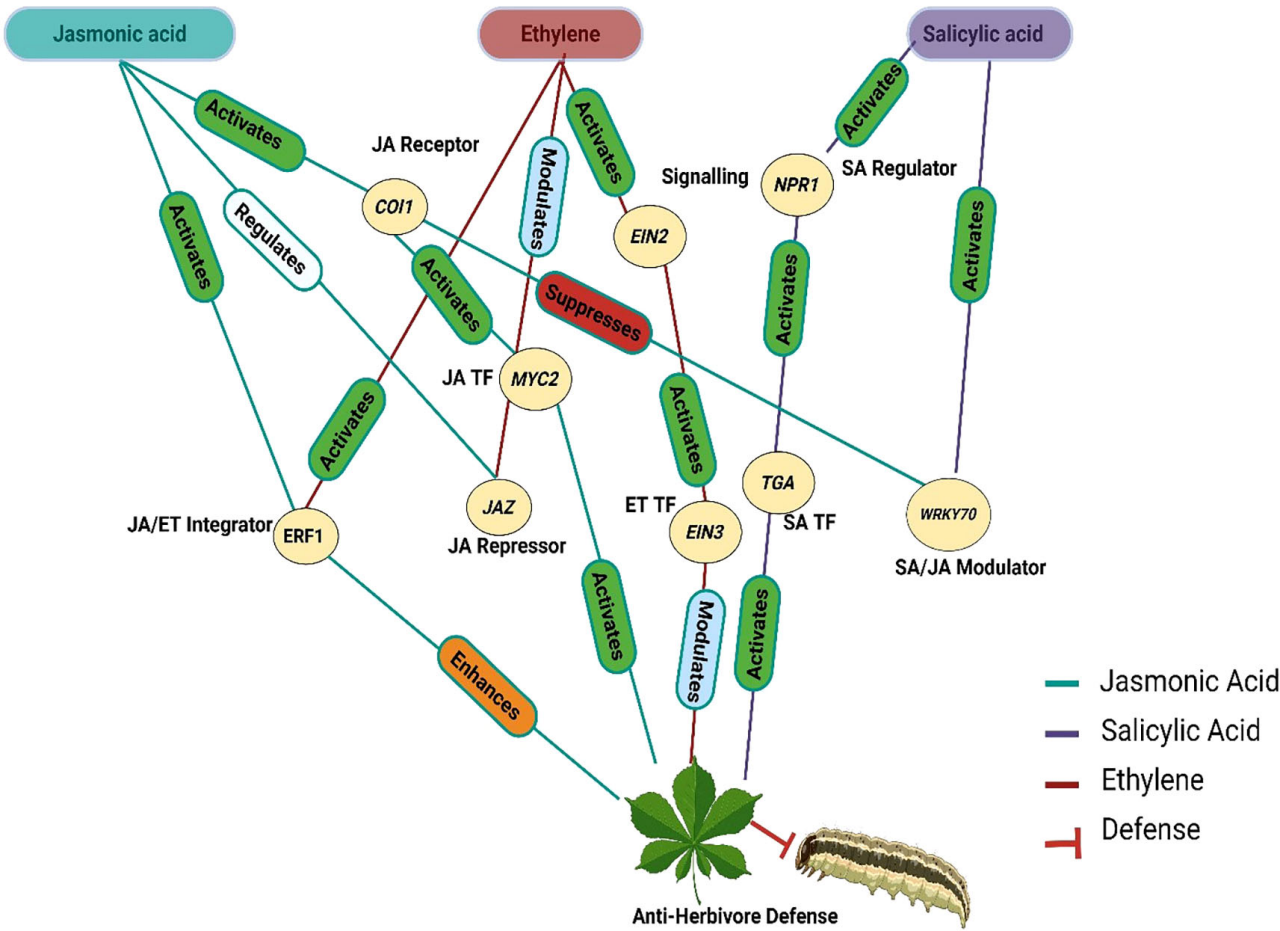
Crosstalk and integration of jasmonic acid (JA), salicylic acid (SA), and ethylene (ET) pathways in defense responses against herbivores. The diagram illustrates the complex signaling interactions among the JA, SA, and ET pathways in mediating plant defenses. The JA pathway initiates defense via COI1 and MYC2, with regulatory control by JAZ repressors. ET signaling interacts synergistically with JA, enhancing defenses via ERF1 activation downstream of JA-ET convergence. SA signaling, regulated by NPR1 and TGA, activates defenses against both herbivores and pathogens, while WRKY70 modulates antagonism between SA and JA pathways. Pathways are color-coded: teal for JA, purple for SA, red for ET, and brown for defense outcomes (created using BioRender.com).

**Figure 8:**
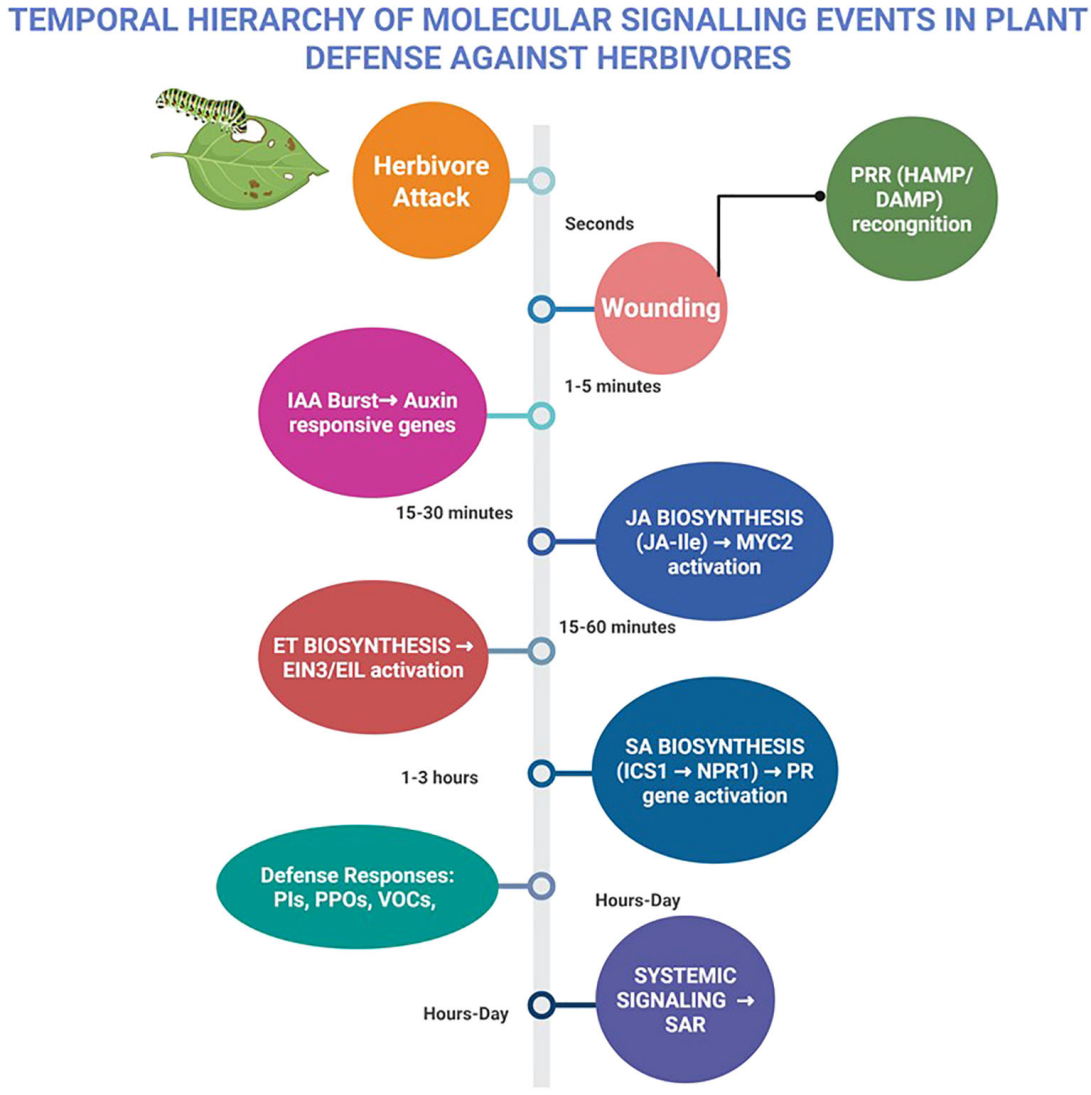
Temporal hierarchy of molecular signaling pathways activated during plant defense against herbivores. Herbivore attack triggers immediate perception of herbivore-associated molecular patterns (HAMPs) and damage-associated molecular patterns (DAMPs) by pattern recognition receptors (PRRs). Within seconds to minutes, early signaling events such as calcium (Ca²^+^) influx, reactive oxygen species (ROS) burst, and MAPK activation are initiated. Indole-3-acetic acid (IAA) accumulates rapidly within 30–60 seconds, peaking around 5 minutes, preceding Jasmonic acid (JA) biosynthesis which activates within 5–30 minutes’ post-attack. Ethylene (ET) signaling synergizes with JA responses within 30–60 minutes, while salicylic acid (SA) signaling becomes prominent at later stages (hours), particularly under phloem-feeding herbivores. These sequential hormone activations drive downstream defense gene expression (protease inhibitors, polyphenol oxidases, secondary metabolites, and volatiles) and systemic acquired resistance (SAR) through long-distance mobile signals. The time frames represent experimentally observed approximate windows based on literature review, (created using BioRender.com).

The integration of JA, SA, ET, and IAA pathways enables plants to dynamically adjust their defense responses. While JA and ET primarily counteract chewing herbivores, SA regulates responses to phloem-feeders and modulates JA-driven defenses through NPR1 and WRKY70 (Lazebnik et al., 2014; Arif et al., 2021; Zafeiriou et al., 2022; Ali et al., 2024b). Importantly, indole-3-acetic acid (IAA) functions as an early systemic signal that precedes jasmonic acid activation upon herbivory. Machado et al. (2016) demonstrated that in *Nicotiana attenuata*, IAA levels rise rapidly within 30–60 seconds after wounding and peak at 5 minutes post-Manduca sexta attack, initiating auxin-responsive gene expression before JA biosynthesis is fully engaged. This early auxin burst independently propagates to distal tissues and modulates JA-dependent secondary metabolism, including phenolamide and anthocyanin biosynthesis, essential for downstream herbivore defense activation. Such rapid auxin signaling interacts with MAPK activation, ROS production, and hormonal crosstalk to fine-tune systemic defense responses (Steppuhn et al., 2004; Li et al., 2022). High-resolution transcriptomic studies reveal rapid transcriptional reprogramming in different plant species within minutes of herbivory (Pandey et al., 2017; Machado et al., 2016; Zafeiriou et al., 2022). In chickpea, Pandey et al. (2017) reported activation of JA and ET networks as early as 20 minutes post-wounding, while suppressing growth-associated hormonal pathways such as auxin and gibberellins. Similar rapid hormonal shifts have been observed in Nicotiana and Arabidopsis, underscoring the importance of temporally synchronized phytohormone crosstalk in tailoring herbivore-specific defense outputs (Montesinos et al., 2024; Kamweru et al., 2022; Vishwanath et al., 2024). These multi-hormonal pathways and regulatory networks equip plants with dynamic, adaptable defenses against diverse herbivore challenges, with integrated JA, SA, ET, and IAA interactions schematically represented in **Figure 7**. To further clarify the dynamic sequence of molecular responses, a temporal model summarizing the rapid perception, early signaling, hormonal activation, defense gene expression, and systemic signaling events triggered during herbivore attack is presented in **Figure 8**.

It is important to emphasize that most mechanistic insights described herein, including hormonal crosstalk, defense activation, and temporal signaling sequences, have been derived from laboratory- and greenhouse-based experiments conducted under controlled environmental conditions, primarily using model systems such as *Arabidopsis thaliana*, *Nicotiana attenuata*, maize, and chickpea. While these studies offer detailed molecular frameworks, additional research is needed to fully validate and scale these mechanisms under field conditions, where environmental variables and complex multi-trophic interactions may influence defense outcomes.

## 3 Physical and chemical defenses in plants

Plants defend themselves against herbivorous insects using preformed structural barriers and inducible chemical weapons. These physical and biochemical traits function in concert with phytohormone-regulated signaling, creating a dynamic, multilayered defense strategy. This section presents a concise synthesis of core structural defenses (e.g., trichomes, waxes, cuticle) and chemical responses (e.g., phenolics, alkaloids, VOCs), highlighting their integration with hormonal pathways such as JA, SA, and ET.

### 3.1 Structural defenses

**Figure 9:**
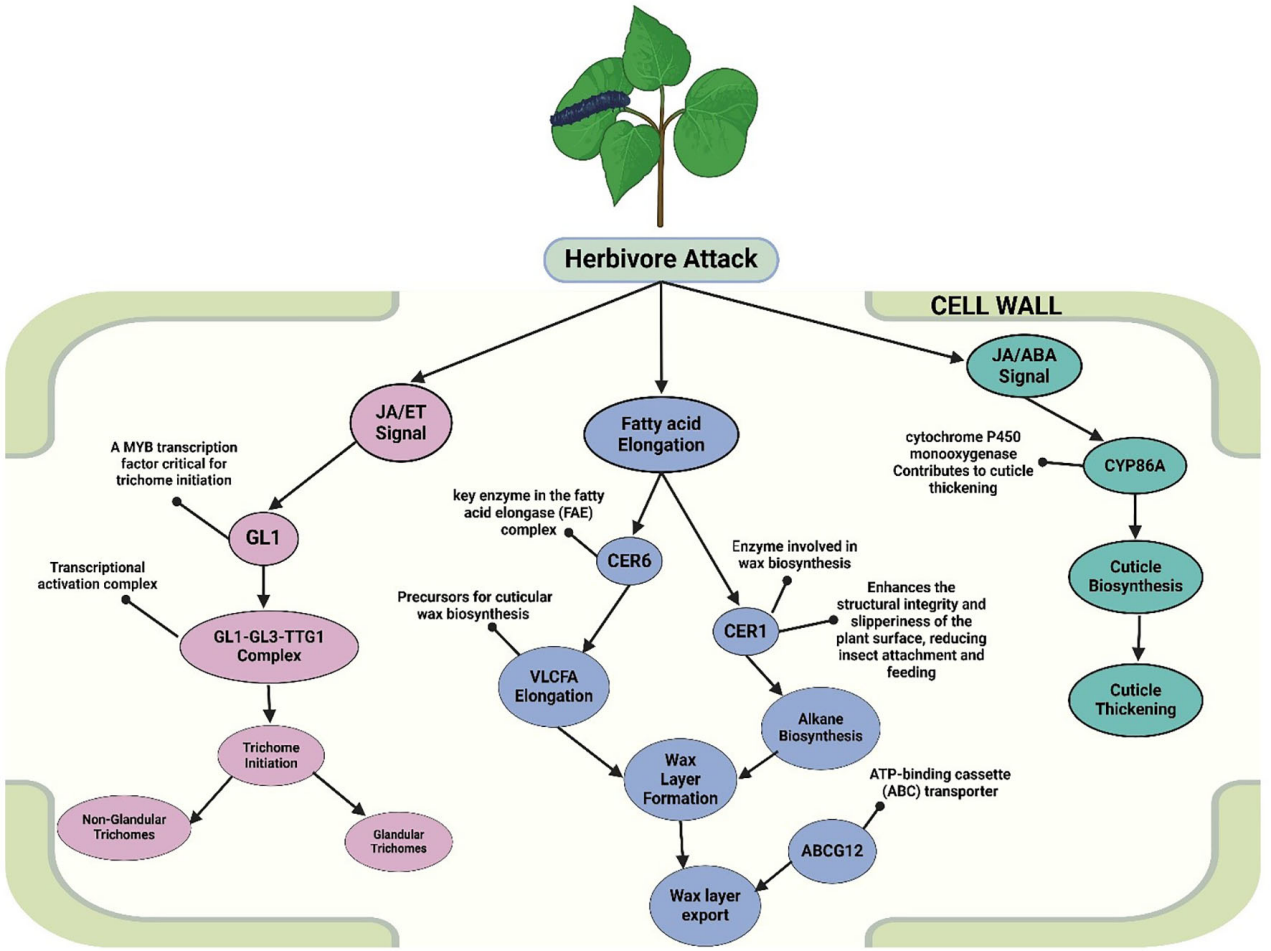
Molecular mechanisms underlying structural defenses of plants against herbivore attack. The figure illustrates the complex molecular mechanisms underlying the structural defenses of plants in response to herbivore attack, emphasizing the roles of trichome development, cuticle thickening, and wax layer formation (created using BioRender.com).

Trichomes, cuticle layers, and waxes act as critical mechanical barriers against herbivory (**Figure 9**). Nonglandular trichomes prevent insect attachment, while glandular trichomes secrete toxic metabolites including terpenoids and alkaloids (Wang et al., 2021a, b; Balaji and Jambagi, 2024). Trichome development is controlled by the GL1–GL3–TTG1 (GL1–GL3–TTG1 complex) and downstream targets like GL2, modulated by feedback (Pattanaik et al., 2014; Pei et al., 2024; Zumajo-Cardona et al., 2023). JA and gallic acid influence trichome density via MYC2, integrating light and wound signals (Brian and Bergelson, 2003). JA–ET crosstalk further enhances glandular secretion and patterning in Arabidopsis through GL3 (Yoshida et al., 2009; Song et al., 2022). Cuticular waxes, composed of Very-Long-Chain Fatty Acids (VLCFAs), alkanes, and esters, minimize desiccation and deter insect feeding (Zeisler-Diehl et al., 2018; Batsale et al., 2021). VLCFAs are derived from C16/C18 fatty acids and elongated in the ER by the FAE complex (Batsale et al., 2023). Export to the surface is mediated by ABC (ATP-Binding Cassette Transporters) such as ABCG12 (CER5), reinforcing the cuticle barrier (Pighin et al., 2004). Wax layers also trap VOCs that repel herbivores or attract predators (Camacho-Coronel et al., 2020; Xue et al., 2017). ABA signaling enhances wax biosynthesis under herbivory (Lewandowska et al., 2024; Joubès and Domergue, 2018).

### 3.2 Chemical defenses

Plants produce diverse chemical compounds including phenolics, alkaloids, terpenoids, VOCs, and protease inhibitors (Divekar et al., 2023a, 2022; Vasantha-Srinivasan et al., 2024). Their synthesis is induced by HAMPs or wounding and regulated by JA and SA signaling (Sharma et al., 2017; Malik et al., 2021; Nguyen et al., 2022). Phenolic compounds such as flavonoids, tannins, and lignins act through multiple mechanisms digestive inhibition, nutrient sequestration, or cell wall reinforcement (Kumar et al., 2020; Singh et al., 2021; Iqbal and Poór, 2024; Balakrishnan et al., 2024). Flavonoids and tannins interfere with enzymes or form indigestible complexes, while lignins strengthen tissue resistance. Alkaloids like nicotine and caffeine disrupt herbivore neural and metabolic pathways (Matsuura and Fett-Neto, 2015; Steppuhn et al., 2004; Garvey et al., 2020; Abernathy et al., 2023; Raisch and Raunser, 2023; Mostafa et al., 2022). Nicotine overstimulates nicotinic receptors; caffeine inhibits phosphodiesterase. Their biosynthesis is JA-dependent, involving Putrescine N-Methyltransferase (PMT) and caffeine synthase (Yang et al., 2016). Terpenoids-monoterpenes, sesquiterpenes, and diterpenes exert toxicity by disrupting membranes, mimicking hormones, or inhibiting neural enzymes (Konuk and Ergüden, 2020; Tsang et al., 2020; Zielińska-Błajet and Feder-Kubis, 2020; Câmara et al., 2024). Diterpenes target mitochondrial function (Yang et al., 2022). Their synthesis is upregulated via the Mevalonate Pathway (MVA) and Methylerythritol Phosphate (MEP) pathways (Opitz et al., 2014; Singh et al., 2024c; Ghorbel et al., 2021). VOCs, especially Green Leaf Volatiles (GLVs) and Herbivore-Induced Plant Volatiles (HIPVs), deter herbivores and attract predators (Mortensen, 2013; Allmann et al., 2013; Ameye et al., 2018; Jones et al., 2022a, b; Zhang et al., 2017; Frago et al., 2022; Matsui and Engelberth, 2022). Hexenal disrupts olfactory cues; methyl jasmonate recruits parasitoids. VOCs also prime systemic defense in neighboring tissues. Protease inhibitors (PIs) and tannins impair digestion by targeting gut proteases and binding proteins (Divekar et al., 2023b; Cid-Gallegos et al., 2022; Molino et al., 2021; Iqbal and Poór, 2024; Mora et al., 2022). JA and SA regulate the expression of key defense genes such as Proteinase Inhibitor II (PI-II) from *S. lycopersicum* and Phenylalanine Ammonia-Lyase (PAL), which is conserved across several species including *Arabidopsis* and *Nicotiana*, providing rapid and localized resistance against herbivores (Farmer and Ryan, 1992). **Table 1** systematically summarizes various induced defense compounds and their specific actions against herbivores.

**Table 1:** Plant metabolites involved in defenses against insect attacks and their modes of action.

Compound(s)	Plant(s)	Attacking insect(s)	Mode of action	Reference
Nicotine Nornicotine Anabasine Anatabine	*Nicotiana tabacum*	*Phthorimaea operculella*	Induced by vibrational signals, deterring pest attack	Pinto et al., 2019
Anthocyanins	*Arabidopsis thaliana*	Lepidopteran insects	Induced by leaf vibrations produced by chewing herbivores, deterring pest attack	Kollasch et al., 2020
Alcohol Aldehyde Hydrocarbon Ketone Ester Benzenoid Terpenoid	*Aquilaria sinensis*	*Heortia vitessoides*	Attracts the insect predator *Cantheconidea concinna*	Qiao et al., 2018
Turpentine α-terpineol Eucalyptol	*Cinnamomum camphora* *Pinus* species	*Plutella xylostella*	Reduces herbivore attack and disrupts mating	Wang et al., 2016
(E)-4,8-dimethyl-1,3,7-nonatriene	*Gossypium hirsutum*	*Spodoptera littoralis*	Suppresses olfactory signaling pathways	Hatano et al., 2015
Coumarins	*Artemisia granatensis*	*Spodoptera littoralis* *Myzus persicae* *Rhopalosiphum padi*	Disrupts herbivore attack on plants	Barrero et al., 2013
3-methyl-3-pentanol 2,5-hexanedione Tetradecanal	*Brassica campestris*	*Spodoptera litura*	Reduces feeding and odor selection under cadmium stress	Guo et al., 2024
(3E)-4,8-dimethyl-1,3,7-nonatriene Caryophyllene Humulene	*Vitis vinifera*	*Tetranychus urticae*	Attracts natural predators that feed on spider mites	Van Den Boom et al., 2004
β-caryophyllene (E)-β-farnesene (E)-4,8-dimethyl-1,3,7-nonatriene	*Vitis vinifera*	*Lobesia botrana*	Attracts grapevine moth females	Tasin et al., 2007
Benzoxazinoids	*Triticum aestivum*	*Rhopalosiphum padi*	Improves plant resistance against insect herbivores in wheat	Shavit et al., 2022
Nonyl tetradecyl ether Hexacosane 2-hexyl-1-decanol Tetratriacontane Heneicosane Octacosane	*Aloe barbadensis*	*Manduca sexta* *Spodoptera frugiperda*	Prevents the feeding of larvae	Johnson et al., 2023
Plumieride	*Himatanthus drasticus*	*Callosobruchus maculatus*	Inhibits intestinal α-amylases and reduces *C. maculatus* infestation	Morais et al., 2021
β-ocimene Thuja-2,4(10)-diene Terpinene	*Brassica oleracea*	*Pieris rapae* *Plutella xylostella*	Attracts natural parasitoids to defend against insect attack	Bruinsma et al., 2009
(E)-β-ocimene	*Phaseolus lunatus*	*Tetranychus urticae*	Increases volatile emission and enhances biological control of spider mites	Menzel et al., 2014
Polyphenol oxidases	*Bouteloua dactyloides*	*Blissus occiduus*	Exhibits antinutritional activity	Heng-Moss et al., 2004
Chitinases	Hybrid of *Populus alba* (white poplar) × *P. tremula* (common aspen)	*Malacosoma disstria*	Exhibits toxicity against larvae	Ralph et al., 2006
Threonine Citric acid Alanine	*Jacobaea aquatica*	*Frankliniella occidentalis*	Inhibits feeding and reduces thrips populations	Wei et al., 2021
Lectins	*Nilaparvata lugens*	*Triticum aestivum*	Exhibits antinutritional activity	Saha et al., 2006
Borneol Eucalyptol (+)-camphor	*Artemisia sieversiana* *A. sylvatica*	*Callosobruchus chinensis*	Chemicals from galls that exhibit insecticidal activity	Liu et al., 2024
Sorbitol Xylitol	*Cajanus platycarpus* *C. cajan*	*Helicoverpa armigera*	Reduces nutrient availability to insects and enhances specific defense hormones and pathways	Dokka et al., 2024
9-hydroxy-10-oxo-12(Z),15(Z)-octadecadienoic acid (9,10-KODA)	*Zea mays*	*Spodoptera frugiperda*	Arrests the growth of fall armyworm larvae, primes the plant for enhanced wound-induced defense gene expression, and modulates GLV signaling for improved resistance	Yuan et al., 2023
1,8-cineole α-pinene Linalool Thymol Carvacrol	*Eucalyptus globulus* *Citrus sinensis* *Mentha arvensis*	*Tribolium castaneum* *Plutella xylostella* *Bemisia tabaci*	Inhibits the growth and disrupts the development of pests and repels pests by disrupting olfactory receptors	Qasim et al., 2024
Quercetin Rutin	*Pyrus ussuriensis* *P. bretschneideri*	*Cydia pomonella* *Grapholita molesta*	Upregulated in response to pest feeding, serving as defense compounds	Zhang et al., 2024b
Cardenolides Iridoid glycosides Furanocoumarins	*Asclepias* species *Plantago* species *Pastinaca sativa*	*Danaus plexippus* Caterpillars and beetles *Papilio polyxenes*	Inhibits sodium-potassium pumps in the pest Converted into reactive compounds that denature defense proteins in insects Binds to DNA, causing toxicity under UV light exposure	Blanchard and Holeski, 2024
BrPGIP3 (polygalacturonase-inhibiting protein)	*Brassica rapa*	*Phaedon cochleariae*	Inhibits polygalacturonases expressed by the leaf beetle, reducing the pest’s ability to hydrolyze pectin in the plant cell wall	Haeger et al., 2020
p-hydroxycinnamic acid	*Pinus* species	*Ips typographus*	Acts as an antifeedant, disrupts digestion, and repels pests.	Latreche and Rahmania, 2011
Nicotine	*Nicotiana* species	*Manduca sexta*	Acts as a neurotoxin, disrupting nervous system function in pests	Howe and Herde, 2015
Indole Methyl anthranilate	*Zea mays*	*Spodoptera exigua*	Emitted by maize in response to maize plant elicitor peptide 3 and attracts parasitoids and deter herbivores	Huffaker, 2015
Indole	*Zea mays*	*Spodoptera exigua*	Primes plant defense responses by enhancing early signaling events, such as MAPK activation	D’Alessandro et al., 2006
(Z)-3-hexenol (E)-2-hexenal	*Zea mays*	*Spodoptera littoralis*	Activates Ca^2+^ flux in plants, triggering early defense response and reducing pest feeding and performance	Farag and Paré, 2002
β-ocimene	*Arabidopsis thaliana*	*Myzus persicae*	Acts as a signal to attract natural enemies of aphids	Fäldt et al., 2003
Linalool	*Medicago truncatula*	*Spodoptera exigua*	Increases PPO activity, making the pest more susceptible to pathogens	Navia-Giné et al., 2009
Methyl salicylate	*Nicotiana tabacum*	*Helicoverpa armigera*	Signals systemic acquired resistance and repels herbivores	Shulaev et al., 1997
α-pinene	*Pinus sylvestris*	*Dendrolimus pini*	Acts as a feeding deterrent and exhibits larval toxicity	Raffa et al., 2005
β-caryophyllene	*Zea mays*	*Diabrotica virgifera*	Attracts entomopathogenic nematodes that parasitize rootworm larvae	Rasmann et al., 2005
Eugenol	*Ocimum basilicum*	*Spodoptera litura*	Exhibits insecticidal activity and disrupts larval feeding	Nerio et al., 2010
Carvacrol	*Origanum vulgare*	*Sitophilus oryzae*	Exhibits fumigant toxicity and disrupts respiratory functions	Kim et al., 2003
(E)-β-farnesene	*Arabidopsis thaliana*	*Myzus persicae*	Acts as an alarm pheromone, repelling aphids and attracting their natural enemies	Pickett et al., 1992
(Z)-3-hexenyl acetate	*Zea mays*	*Spodoptera littoralis*	Attracts parasitoid wasps, enhancing indirect plant defense mechanisms	Turlings et al., 1995
Methyl jasmonate	*Nicotiana attenuata*	*Manduca sexta*	Induces the production of nicotine and other defense compounds, deterring herbivory	Baldwin, 1998
(E)-4,8-dimethyl-1,3,7-nonatriene	*Phaseolus lunatus*	*Tetranychus urticae*	Attracts predatory mites, reducing herbivore populations	Arimura et al., 2000
(E,E)-α-farnesene	*Glycine max*	*Helicoverpa zea*	Attracts parasitic wasps, facilitating the biological control of herbivores	Röse and Tumlinson, 2004
(E)-β-ocimene	*Medicago truncatula*	*Spodoptera exigua*	Serves as a signal to attract natural enemies of herbivores	Leitner et al., 2005

## 4 Insect counter-defense mechanisms

**Figure 10:**
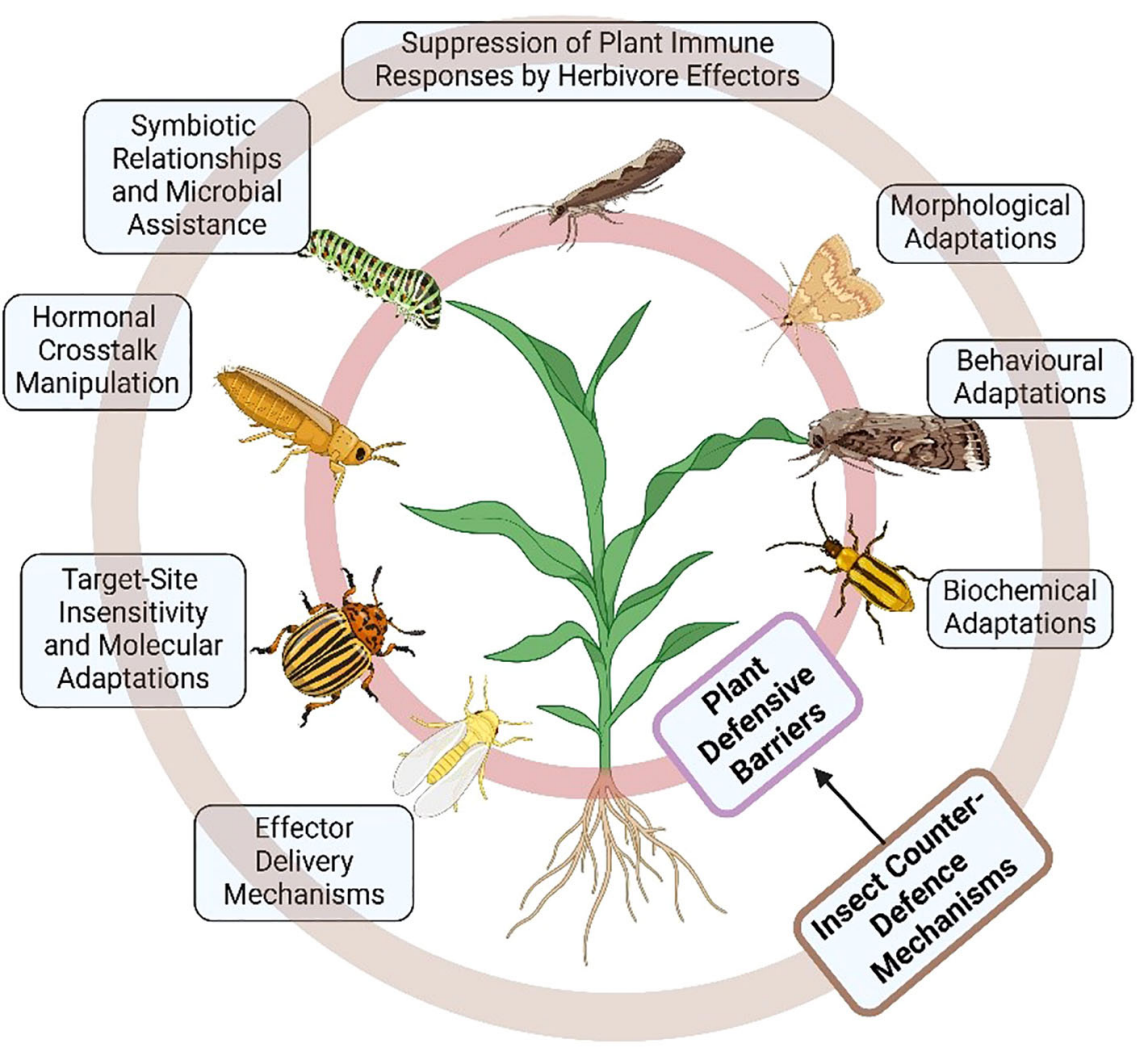
A schematic representation of insect counter-defense mechanisms against plant defense barriers. These include morphological, behavioral, and biochemical adaptations, in addition to effector delivery mechanisms, hormonal crosstalk manipulation, and the suppression of plant immune responses by herbivore effectors. Additional strategies involve target site insensitivity, molecular adaptations, and symbiotic relationships that enhance insect survival against plant defenses (created using BioRender.com).

Herbivorous insects have evolved precise and multi-layered strategies to overcome plant immune responses. These counter-defenses are not merely structural or behavioral but deeply integrated at the molecular and hormonal levels, allowing insects to exploit host vulnerabilities and manipulate plant immunity. Below, we elaborate the most mechanistically relevant counter-strategies insects use to suppress, evade, or reprogram plant defense networks (**Figure 10**).

### 4.1 Behavioral adaptations

Herbivores engage in finely tuned behaviors that limit exposure to inducible plant defenses. For example, leaf miners such as *Liriomyza* spp. feed internally, avoiding detection by external pattern recognition receptors and minimizing activation of systemic hormonal cascades (Hamza et al., 2023). Gall-inducing insects hijack developmental signaling to create nutrient-rich microenvironments shielded from chemical defenses (Mishra et al., 2024b). Additionally, many insects exploit phenological windows targeting young, less lignified tissues with lower concentrations of phenolics and VOCs (Milton, 1979). Physical adaptations, such as hydrophobic tarsal pads in thrips and beetles, allow navigation across resinous or trichome-dense surfaces, mitigating mechanical restriction and enhancing feeding efficiency (Voigt et al., 2017).

### 4.2 Target site insensitivity and molecular adaptations

At the molecular level, insects have developed specific mutations and regulatory mechanisms to resist plant defenses. Resistance to plant toxins often arises from genetic mutations that can alter the target sites of these compounds (Petschenka and Dobler, 2009). Some insects, such as milkweed bugs and monarch butterflies, exhibit remarkable adaptations through mutations in the sodium–potassium ATPase gene. These mutations reduce the binding affinity of cardenolides, which are toxic steroids produced by milkweed plants, to the enzyme and effectively neutralize their inhibitory effects. This molecular modification enables these insects to not only tolerate high levels of cardenolides but also sequester these compounds for use as a chemical defense against predators (Aardema et al., 2012). In the Colorado potato beetle *L. decemlineata*, the production of digestive enzymes, including lipases and cellulases, is upregulated to break down structural components of plants, such as waxes and cellulose. This enzymatic plasticity helps in coping with different plant species or varying environmental conditions (Wilhelm et al., 2024).

### 4.3 Suppression of plant immune responses by herbivore effectors

In their arms race with plants, herbivorous insects have evolved the ability to suppress plant immune responses by using specialized proteins known as effectors. These molecules, secreted in the saliva or other oral secretions of insects, can directly interfere with the immune signaling pathways of plants, enabling successful colonization and feeding (Wang et al., 2023). Herbivores use effectors to manipulate plant immune signaling systems, such as those regulated by JA, SA, and ET. These phytohormones orchestrate plant defense responses against different types of attackers (Caarls et al., 2015). The primary goal of herbivore effectors is to suppress recognition by plants and prevent the downstream activation of these pathways. Herbivorous insects secrete effectors that suppress plant PTI, which is activated upon recognition of HAMPs, thereby facilitating successful feeding (Basu et al., 2018). For example, *Helicoverpa zea* secretes glucose oxidase (GOX), which disrupts ROS signaling in the plant host, weakening defense activation (Tian et al., 2012), and aphids deliver salivary effectors to inhibit R-proteins and suppress ETI cascades (Elzinga et al., 2014). Some insects, like weevils, modulate polygalacturonase inhibitors to suppress cell wall-based defenses, facilitating feeding with minimal resistance (Kalunke et al., 2015; Gong et al., 2023). Through such diverse adaptations, herbivorous insects effectively navigate plant defenses. Understanding these mechanisms is vital for developing innovative pest management strategies in agricultural systems.

### 4.4 Hormonal crosstalk manipulation by herbivores

Herbivorous insects can manipulate plant hormonal crosstalk to circumvent defenses by exploiting the antagonistic interaction between JA and SA. Aphids and whiteflies stimulate SA accumulation while suppressing JA-mediated defenses, resulting in reduced synthesis of JA-regulated compounds such as PIs and secondary metabolites (Zarate et al., 2007; Zhang et al., 2013; Xu et al., 2019). This hormonal manipulation facilitates phloem feeding, thereby promoting insect colonization and reproduction (VanDoorn et al., 2015; Zhang et al., 2018). Whiteflies (*Bemisia tabaci*) secrete salivary effectors to activate SA signaling, dampening JA-mediated defenses in host plants like tomato (Zhang et al., 2018), and aphids use similar strategies, activating SA and suppressing JA to weaken defenses, facilitating efficient feeding (Zhang et al., 2023a; 2024c). The tobacco hornworm (*Manduca sexta*) secretes GOX to interfere with the oxidative burst associated with JA signaling, reducing overall plant defense and enhancing feeding efficiency (Bari and Jones, 2009). These examples illustrate the intricate strategies by which herbivorous insects manipulate plant hormonal crosstalk, thereby enhancing their ability to overcome plant defenses.

Insect herbivores have evolved intricate countermeasures to overcome plant defenses mediated by ABA, a key hormone involved in stress adaptation (Park et al., 2019). One such strategy involves the secretion of salivary effector proteins that disrupt ABA signaling to suppress plant defensive responses. These effectors may target crucial components of the ABA pathway, including ABA receptors (PYR/PYL/RCAR), protein phosphatases (PP2Cs), or transcriptional regulators, effectively modulating guard cell behavior and secondary metabolite production (Korek and Marzec, 2023). Such interference can compromise stomatal closure, leading to enhanced water loss and weakened physical barriers, ultimately increasing insect feeding efficiency. Additionally, certain insect salivary proteins have been shown to mimic phosphatases, potentially dephosphorylating key signaling proteins involved in ABA cascades, thereby dampening the transcription of ABA-responsive genes that are otherwise critical for defense reinforcement under combined drought and herbivore pressure (Khan et al., 2015). Beyond signaling disruption, insects may enzymatically degrade or detoxify ABA through metabolic conversion pathways, reducing the hormone’s bioavailability. Some evidence suggests that insect species may upregulate specific oxidases or transferases that modify plant-derived ABA into inactive forms (Kosma et al., 2009). While such detoxification pathways remain underexplored, they represent a compelling frontier in plant-insect interaction research. Moreover, plant biotechnology research suggests that enhancing ABA pathway robustness through genetic engineering can mitigate such insect manipulations. For instance, transgenic lines with fortified ABA signaling components have shown improved resilience to both abiotic and biotic stress, although precise gene targets and field validation remain critical (Dhariwal et al., 1998). These multifaceted counter-adaptations reflect the dynamic co-evolution between plants and insect herbivores, underscoring the need for integrated pest management strategies that consider both plant resistance and insect plasticity in manipulating defense signaling networks.

### 4.5 Effector delivery mechanisms in herbivores

Herbivorous insects have evolved precise delivery systems to deploy effector molecules that interfere with host immunity at the cellular and molecular levels. Piercing–sucking insects, including aphids and whiteflies, utilize slender stylets to navigate intercellular spaces and deliver salivary effectors directly into the cytoplasm of phloem and mesophyll cells, where they disrupt host immune signaling (Wang et al., 2023; Naalden et al., 2021). For instance, *Myzus persicae* secretes Mp10, which suppresses callose deposition at sieve plates, thereby maintaining phloem conductivity for sustained nutrient uptake (Bos et al., 2010). *Bemisia tabaci* releases the effector BtE1 that interferes with SA-mediated defense cascades, leading to reduced expression of defense-related genes and enhanced phloem extraction efficiency (van Kleeff et al., 2024). Similarly, rice planthoppers like *Nilaparvata lugens* translocate effectors such as NlNSE1 and NlNSE2 into host tissues to suppress JA biosynthetic and downstream signaling pathways, thereby diminishing the accumulation of phenolic and flavonoids essential for herbivore deterrence (Lou et al., 2005). These strategies facilitate long-term colonization and reproductive success. Chewing insects, such as caterpillars and coleopterans, also employ salivary effectors during feeding to suppress localized immune responses. *Helicoverpa armigera* secretes GOX, which attenuates the oxidative burst by downregulating NADPH oxidase activity and interfering with ROS-dependent amplification of JA signaling (Tian et al., 2012). Likewise, *L. decemlineata* produces polygalacturonase (LDPG1), which degrades homogalacturonan in the plant cell wall matrix, thereby weakening structural integrity and facilitating herbivore feeding (Gosset et al., 2009). Other herbivores have evolved enzymatic adaptations that modulate secondary metabolite activation. *N. lugens* secretes β-glucosidases that hydrolyze glucosylated precursors, preventing the activation of toxic glucosinolates and reducing defense metabolite pools (Wang et al., 2008). Similarly, sawflies feeding on Brassicaceae manipulate the glucosinolate–myrosinase system to suppress the release of isothiocyanates, diminishing plant chemical deterrence (Ahuja et al., 2011). The diversification of effector repertoires across insect taxa illustrates a sophisticated evolutionary response to host immunity, reflecting the coevolutionary pressure exerted by plant surveillance systems. While plants continuously evolve novel receptors and immune modulators to recognize and neutralize insect effectors, herbivores reciprocally fine-tune effector specificity, expression timing, and delivery routes to evade detection and maintain feeding success (Wang et al., 2023). Understanding these dynamic molecular dialogues offers promising avenues for engineering crops with enhanced recognition capacity or effector-triggered resistance, laying the foundation for next-generation pest management strategies.

## 5 Molecular crosstalk between plants and insects

### 5.1 Signaling molecules in plant–insect interactions

The intricate interplay between plants and herbivorous insects involves signaling molecules and genes orchestrating both plant defenses and insect counterstrategies (Zebelo and Maffei, 2015; Pang et al., 2021). The JA derivative JA-Ile, in particular, is central to plant defenses against chewing insects. It binds to the COI1–JAZ receptor complex, degrading JAZ repressors and activating transcription factors like MYC2, which, in turn, induces PIs and secondary metabolites, such as glucosinolates and alkaloids (Kumar et al., 2024). SA plays a pivotal role in plant defense against phloem-feeding insects by activating PR genes through the SA signaling pathway (Fang et al., 2025). Systemin and ET amplify local and systemic defenses by interacting with the JA and SA pathways, while VOCs further enhance resistance (Erb, 2018). Insect-derived elicitors, or HAMPs, refine plant responses. For instance, fatty acid–amino acid conjugates from *S. frugiperda* and β-glucosidase from *Pieris brassicae* activate MAPK cascades via plant LRR-RLK receptors, boosting secondary metabolite production (Vidhyasekaran, 2016). In contrast, insect salivary effectors such as aphid Mp55 suppress plant defenses by reducing the accumulation of defense-related compounds, thereby facilitating infestation (Elzinga et al., 2014). In addition to Mp55, several candidate salivary effectors have been identified from *M. persicae*, including Mp10, Mp42, and MpC002, which are predicted to interfere with plant immune responses (Bos et al., 2010). Rapid plant defense signaling involves ROS and calcium ion (Ca²^+^), which activate transcription factors like WRKY through MAPK and CDPK pathways, further amplifying stress-responsive gene expression (Adachi et al., 2015). Additionally, jasmonate signaling activates MYC transcription factors, such as MYC2, to regulate defense responses (Lorenzo et al., 2004).

### 5.2 Role of microRNAs and small interfering RNAs in mediating plant–insect interactions

Small RNAs, including miRNAs and siRNAs, regulate plant defenses by fine-tuning gene expression post-transcriptionally. Both miR393 and miR319 enhance JA defenses by suppressing auxin signaling and modulating JA biosynthesis, promoting secondary metabolite production (Schommer et al., 2008; Iglesias et al., 2014; Jacob et al., 2021), and siRNAs, such as phasiRNAs derived from miRNA-targeted NLR transcripts, silence genes that negatively regulate JA signaling, ensuring resource-efficient defenses during herbivore attacks (Liao et al., 2022). Cross-kingdom RNA transfer adds complexity to plant-insect interactions. Plants can deliver small RNAs via extracellular vesicles to insects, targeting genes involved in detoxification or digestion, such as cytochrome P450s in *H. armigera*, thereby disrupting insect physiology (Zhao et al., 2024). Conversely, *H. armigera* miRNAs, such as miR854, manipulate plant defenses by targeting JA-signaling regulators like WRKY, shifting the JA–SA balance to weaken resistance (Tan et al., 2012; Chen et al., 2019a). Small RNAs secreted by insect saliva can target key plant defense genes, including those involved in lignin biosynthesis (e.g., MYB transcription factors), RLK signaling pathways, and ROS generation, thereby attenuating both structural and biochemical defenses (Han et al., 2025). For example, siRNAs from aphids and whiteflies interfere with NADPH oxidases, reducing the oxidative bursts crucial for secondary metabolite production (Hu et al., 2020). These RNA-mediated interactions highlight the sophistication and complexity of the co-evolutionary arms race between plants and herbivores.

## 6 Biotic factors influencing plant–insect interactions

Biotic factors, including symbiotic microbes, endophytes, and natural enemies, shape plant–insect dynamics by mediating ecological and molecular interactions that enhance plant resilience to herbivory (Pineda et al., 2013). Microbes, such as mycorrhizal fungi and nitrogen-fixing bacteria, prime hormonal pathways and bolster secondary metabolite production, while endophytes induce systemic resistance and produce bioactive compounds that deter herbivores (Grabka et al., 2022). Plant-associated microbiomes also modulate VOC emissions that attract herbivore predators, reinforcing defense strategies (Raza et al., 2021). Additionally, natural predators and parasitoids not only directly suppress pest populations but also indirectly influence plant immunity through trophic cascades, reinforcing plant defense strategies (Simberloff, 2011).

### 6.1 Role of symbiotic microbes in plant immunity to insect herbivores

Symbiotic microbes critically influence plant–insect dynamics by either enhancing plant immunity or facilitating herbivore adaptation. In the rhizosphere, arbuscular mycorrhizal fungi (AMF) and nitrogen-fixing rhizobia prime plant defenses by modulating phytohormonal pathways. AMF enhance JA-dependent synthesis of terpenoids and phenolics that deter insect feeding (Sharma et al., 2017; Boyno et al., 2023). Sinorhizobium meliloti, which forms nodules in legumes like Medicago truncatula, not only improves nitrogen status but also strengthens aphid resistance through JA-mediated induction of deterrent metabolites (Pandharikar et al., 2020). Endophytic fungi and bacteria within plant tissues also contribute to insect resistance. Fusarium solani-derived endophytes in rice upregulate phenolic biosynthesis and PR gene expression, reducing stem borer infestation (De Lamo and Takken, 2020; Xia et al., 2022). Similarly, Epichloë fungi in grasses produce defensive alkaloids—peramine and lolines regulated by JA, SA, and ET signaling crosstalk (Bharadwaj et al., 2020). Recent work has shown that plant-associated microbiomes directly modulate hormone-regulated defenses in plant–insect interactions (Théatre et al., 2021). A meta-analysis revealed that inoculation with PGPR (e.g., *Pseudomonas fluorescens*, *Bacillus subtilis*) enhances resistance to chewing insects by inducing JA- and ET-mediated defense responses, including elevated PIs and phenolic accumulation in leaves demonstrated under greenhouse conditions in cabbage and maize (Ruiz-Santiago et al., 2025). Endophytic *Trichoderma asperellum* M2RT4 induces systemic resistance against *Tuta absoluta* in tomato by activating both SA and JA signaling pathways and altering volatile emissions to reduce oviposition and larval survival (Agbessenou et al., 2022). Moreover, Root herbivory by insects alters rhizosphere microbial communities, which feeds back to influence aboveground plant defense via ISR-like mechanisms (Friman et al., 2021). These studies highlight direct and indirect hormone-pathway modulation by microbes, contextualized in eco-physiological setups. Additionally, microbes appear to subtly influence IAA- and JA-hormone balance: PGPR-induced auxin changes may prime downstream defense cascades (root-shoot signaling), aligning with the timing and strength of systemic responses (Rashid and Chung, 2017). It is important to emphasize that these effects, though robust in controlled environments, vary significantly with plant genotype, microbial consortia, environmental factors, and insect feeding strategies (Tronson and Enders, 2025). These examples illustrate how microbial partnerships facilitate plant defense suppression via detoxification, hormonal modulation, and nutritional support.

### 6.2 Role of natural predators and parasitoids in modulating plant immunity

Natural predators and parasitoids regulate herbivore populations, indirectly enhancing plant immunity through trophic cascades. By reducing herbivore pressure, they allow plants to allocate resources toward growth and reproduction, making predator–prey interactions important to sustaining plant health (Silliman and Angelini, 2012). Predators like lady beetles (Coccinellidae) prey on aphids, reducing aphid populations and thereby diminishing the secretion of salivary effectors that suppress plant defenses. This predation enables plants to maintain their natural immune responses (Elzinga and Jander, 2013). Parasitoids, such as *Trichogramma* species, parasitize pest eggs and disrupt the host’s ability to produce salivary effectors, similarly reducing herbivore-induced plant-defense suppression and allowing stronger immune activation (Martel et al., 2021). Plants also detect insect oviposition and initiate defenses against subsequent herbivory (Wang et al., 2021c). In *A. thaliana*, for example, oviposition by *P. brassicae* activates an SA-dependent signaling pathway, inducing PR protein expression and enhancing systemic resistance (Gouhier-Darimont et al., 2013). This response involves the recognition of egg-associated elicitors, similar to PAMPs, triggering localized and systemic defense mechanisms to prepare for future attacks.

## 7 Biotechnological and genetic engineering approaches to enhancing plant immunity

The integration of biotechnology with plant immunity research has revolutionized pest-resistant crop development by enabling precise manipulation of molecular defense networks (Klümper and Qaim, 2014). Genetic engineering platforms, including transgenic expression systems, CRISPR/Cas9-mediated genome editing, and RNAi, now allow targeted modulation of phytohormone signaling, transcriptional regulators, and small RNA pathways to strengthen plant immune responses. For instance, transgenic crops expressing *B. thuringiensis* (Bt) genes such as Cry1Ac and Cry1Ab (Crystal Protein) produce δ-endotoxins that bind to cadherin-like receptors in the midgut of lepidopteran pests, leading to pore formation, osmotic imbalance, and cell lysis (Chakrabarty et al., 2022). Overexpression of *Arabidopsis thaliana* Cystatin 1 (AtCYS1), a cystatin gene, enhances resistance to herbivory in Arabidopsis by inhibiting digestive cysteine proteases in insect midguts (Belenghi et al., 2003). However, due to rapid pest adaptation, recent strategies emphasize multigene stacking, such as combining protease inhibitors and lectins, for broader and more sustainable defense (Belenghi et al., 2003).

CRISPR/Cas9 genome editing enables high-precision modification of immune-related loci (Xuebo et al., 2023). Knockout of susceptibility (S) genes like MLO (Mildew Locus O) in barley or DMR6 (Downy Mildew Resistant 6) in tomato and sweet basil has been shown to confer enhanced resistance without growth penalties (Thomazella et al., 2021). Editing key transcriptional regulators like MYC2, MYC3, and MYC4 amplifies JA-responsive pathways and increases the production of proteinase inhibitors and alkaloids, improving resistance against chewing herbivores such as *S. littoralis* (Fernández-Calvo et al., 2011). More recent innovations use dead Cas9 (dCas9) fused to activator domains for transcriptional reprogramming of defense genes, enabling non-mutagenic but inducible defense expression (Gao, 2021).

Host-induced gene silencing (HIGS) leverages RNAi by allowing plants to produce dsRNAs that target essential genes in insect pests upon ingestion. Transgenic tomato and tobacco expressing dsRNAs targeting Helicoverpa armigera genes such as V-ATPase, chitin synthase, and CYP6B6 reduce larval growth and midgut function (Jin et al., 2015; Mamta et al., 2016). Moreover, insects deploy cross-kingdom effectors such as miR29b, which, when delivered via saliva, silence host genes like BAG4 through AGO1 recruitment, impairing defense (Han et al., 2023). Counteracting such miRNAs by designing target mimics or CRISPR editing of AGO1-regulated promoters offers new resistance pathways. Additionally, silencing insect miRNAs like miR-7-5p derepresses OsbZIP43 in rice, activating defense transcription (Zhang et al., 2024d).

However, RNAi-based resistance strategies face critical challenges, including instability of dsRNA in field conditions, limited uptake in phloem-feeding pests, and inconsistent efficacy due to rapid degradation by insect gut nucleases. To overcome these issues, chloroplast genome engineering has been proposed as a transgene containment strategy and a sustainable expression platform for dsRNAs. For instance, Bulle et al. (2023) demonstrated that engineering the chloroplast genome can produce high levels of stable dsRNA, minimizing off-target movement and enhancing pest-specific toxicity, especially for *Scirtothrips dorsalis* (chili thrips).

Metabolic engineering is another frontier, enabling redirection of central metabolism toward defense metabolite production (Tilkat et al., 2024). Overexpression of TPS10 and TPS21 increases emission of volatile monoterpenes such as α-pinene and (E)-β-ocimene, which repel pests or attract their natural enemies (Wang et al., 2021c). Activation of transcription factors like MYB20, MYB85, and WRKY45 enhances flavonoid and lignin biosynthesis, reinforcing physical barriers and modulating ROS homeostasis (Bahrini et al., 2011; Geng et al., 2020).

Advanced synthetic biology approaches integrate multiplex CRISPR editing with hormone-responsive synthetic promoters and field-deployable delivery tools (Vitorino, 2024). For example, star polycation (SPc) nanocarriers improve delivery and stability of dsRNAs or miRNAs, enabling RNAi-mediated pest control in open-field conditions (Abdelrahman et al., 2021). Recently identified compact genome editors such as TnpB, a minimalist RNA-guided endonuclease, offer potential for lightweight editing systems compatible with large-genome crops (Karvelis et al., 2021). Synthetic inducible promoters responsive to pest-associated cues can also be coupled to immune signaling genes, activating defense only under attack to conserve energy (Yang et al., 2022). These molecularly informed strategies exemplify the integration of genome engineering, epigenetic regulation, and metabolic reprogramming for developing pest-resilient crops tailored to dynamic agro ecological challenges (Zaidi et al., 2020; Lyu et al., 2021).

## 8 Challenges and future directions

### 8.1 Gaps in our understanding of plant immunity to insect herbivores

Despite advancements, critical gaps remain in understanding the complexity of plant immunity to herbivores. Hormonal crosstalk between JA, SA, and ET pathways under field conditions, where biotic and abiotic stresses co-occur, is not fully elucidated (Ku et al., 2018; Ament et al., 2010), and trade-offs in JA-SA antagonism, dynamically modulated by herbivore pressures, environmental fluctuations, and genotype-specific regulatory networks, continue to complicate precise predictions in defense allocation (Samanta and Roychoudhury, 2024). Also, the roles of resistance genes, miRNAs, and Long non-coding RNAs (lncRNAs) in herbivore defense are largely unexplored and require functional studies to reveal their precise behaviors (Huang et al., 2023). Newly identified herbivore effectors, such as those found in *P. rapae* and *M. sexta*, demonstrate their ability to manipulate plant defenses, yet their mechanisms and targets need deeper investigation. Additionally, the temporal dynamics of defense activation and specificity under multi-herbivore attacks remain poorly understood (Croy et al., 2021). Addressing these gaps demands integrative approaches that incorporate ecological conditions, coevolutionary pressures, and pest adaptation mechanisms.

In field production systems, plant defense mechanisms operate alongside and often interact with common agronomic practices such as chemical applications and IPM. While agrochemicals (e.g., synthetic insecticides) are effective in reducing pest pressures, they can disrupt hormonal signaling, harm non-target organisms, and promote resistance (Zhou et al., 2024; Ahmad et al., 2024). Conversely, IPM strategies that combine monitoring, biological control, cultural practices, and targeted chemical interventions can support natural plant defense pathways while reducing reliance on pesticides, though adoption and implementation remain highly context-dependent due to economic and logistical challenges (Grasswitz, 2019; Wyckhuys et al., 2023). Incorporating discussions on these practical challenges is essential for aligning mechanistic insights with real-world crop protection, ensuring that laboratory-based discoveries translate effectively into field-resilient plant immunity.

Pest adaptation, a significant impediment in plant protection, involves evolutionary shifts that undermine the long-term efficacy of biotechnological interventions. For instance, *B. thuringiensis* (Bt) cotton, initially celebrated for its effectiveness in reducing lepidopteran pest infestations in India, has increasingly faced challenges due to the development of resistant pest populations under continuous selection pressure (Karimi et al., 2012; Xing and Wang, 2024). This resistance emergence underscores the necessity for robust resistance management strategies such as refuge planting and gene pyramiding to maintain the sustainability of Bt technologies (Bravo et al., 2015). Concurrently, the ecological implications of these interventions require comprehensive scrutiny. The deployment of biocontrol agents and their derivatives, aimed at suppressing pest populations below economic thresholds, contributes to maintaining ecosystem equilibrium by preserving beneficial arthropods (Patil et al., 2021). However, realizing the full potential of such biotechnological tools necessitates integrative frameworks that consider agroecological complexities. While initial field deployments like Bt cotton demonstrated reduced pesticide reliance and increased yield (Sánchez et al., 2018; Singh et al., 2019), challenges such as RNAi variability under field conditions and poor farmer access to information persist (Ramírez-Pool et al., 2024; Shields et al., 2018). The broader shift toward environmentally benign practices, aligned with green chemistry principles, emphasizes reduced toxicity, target specificity, and biodegradability, supporting IPM strategies. Nonetheless, the continued use of synthetic pesticides raises environmental and public health concerns, with mounting evidence of their contribution to soil, water, and air pollution and their bioaccumulative impacts on biodiversity and human health (Lahlali et al., 2022; Antoszewski et al., 2022). Ultimately, translating laboratory innovations into sustainable field solutions will require not only adaptive resistance management and regulatory coherence but also farmer-centric knowledge dissemination and ecosystem-based monitoring for long-term agricultural resilience.

### 8.2 Ethical and ecological considerations for engineering plant immunity

Despite their precision, the deployment of biotechnological tools, such as CRISPR/Cas9 and RNAi, raises ethical and ecological concerns. Genetically modified plants with enhanced resistance may disrupt natural pest–predator dynamics and affect nontarget species via unintended RNAi effects (Lundgren and Duan, 2013; Diaz et al., 2025). Public apprehensions about GM crops, as seen with Bt brinjal in India and stringent GM organism policies in the EU, emphasize the need for transparent risk assessments and stakeholder engagement (Singh, 2018; European Commission, 2024). Ecological concerns, including pest adaptation, gene flow to wild relatives, and the disruption of plant–microbe interactions, necessitate rigorous long-term studies (Mandal et al., 2020). Strategies integrating genetic engineering with agroecological practices can mitigate environmental impacts and foster sustainable pest management (Anderson et al., 2019). Additionally, robust governance frameworks and ecological risk assessments are critical for deploying engineered plants ethically and sustainably, ensuring their role in climate-resilient agriculture while preserving ecosystem integrity (Hilbeck et al., 2011).

Recent changes in regulatory landscapes have started to differentiate genome-edited crops from conventional GMOs. For example, countries like the US, Brazil, and Japan have streamlined regulations for CRISPR-based edits that do not introduce foreign DNA, considering them equivalent to conventional breeding outcomes (EFSA Panel on Genetically Modified Organisms, 2010; Anderson et al., 2019). In contrast, the European Union continues to apply stringent GMO regulations to genome-edited plants, limiting their adoption and research potential (Voigt, 2023). These discrepancies influence global trade, technology diffusion, and food security policy, highlighting the urgent need for harmonized international biosafety standards.

Furthermore, climate change amplifies the complexity of these challenges. Elevated CO_2_ levels, extreme weather patterns, and altered pest pressures may unpredictably interact with transgenic traits, affecting efficacy and stability (Liu et al., 2020). For instance, RNAi-based insecticidal crops may exhibit variable gene silencing efficiency under fluctuating temperatures, potentially compromising pest control and increasing resistance risk (Fletcher et al., 2020). Additionally, CRISPR-driven traits targeting susceptibility (S)-genes may influence unintended pathways under abiotic stress, necessitating context-specific ecological modeling before field deployment. To address these emerging concerns, a new paradigm of “precautionary innovation governance” is recommended (Nascimento et al., 2023). This includes public–private collaborations, real-time monitoring of gene flow, off-target effects, and ecosystem-level feedback mechanisms. Implementing gene-drive containment strategies, temporal deployment limits, and trait-reversal mechanisms (e.g., CRISPR-off switches) can provide adaptive safety controls while ensuring continued innovation (Pawluk et al., 2016). Lastly, multi-stakeholder dialogue involving farmers, ecologists, ethicists, and regulators is essential to develop trust and social license for genome-edited agricultural solutions (Lindberg et al., 2023).

## 9 Concluding remarks

The dynamic interplay between plant immunity and insect herbivores underpins sustainable crop protection and ecological stability. Recent progress in deciphering defense signaling networks including JA-SA crosstalk, volatile-mediated tritrophic interactions, and secondary metabolite biosynthesis has laid a molecular foundation for minimizing pesticide dependency. Emerging tools such as RNA interference (RNAi) and CRISPR/Cas9 offer precision-based modulation of pest-responsive genes, enabling the development of cultivars with tailored immunity to herbivore pressures. However, for field efficacy, future research must integrate metabolomics with spatially distributed field trials to identify defense biomarkers under variable environmental conditions and herbivore pressures. Specifically, CRISPR-edited crops targeting herbivore effector recognition or hormone biosynthesis nodes like JAZ repressors or WRKY transcription factors should be tested in climate-stressed agroecosystems to ensure durability and yield neutrality. Concurrently, multi-omics profiling of plant–microbe–insect interactions, especially involving endophytes, gut microbiota, and rhizosphere consortia, will be vital to unravel context-specific immunity triggers. Integrative strategies combining genome editing, AI-driven phenotyping, and ecological practices such as intercropping and push–pull systems will be instrumental in crafting next-generation climate-resilient crops. Moving forward, transdisciplinary collaboration between molecular biologists, ecologists, agronomists, and data scientists is imperative to translate laboratory innovations into robust field applications that safeguard biodiversity, ensure long-term pest resistance, and secure global food systems amid escalating climate challenges.

## Supplementary material

Supplementary Figure 1 Differentiation between damage-associated molecular pattern (DAMP) and herbivore-associated molecular pattern (HAMP) pathways during plant defense against insect attack. The chart illustrates the distinct pathways and mechanisms of DAMPs and HAMPs in triggering plant defense responses upon insect feeding (created using BioRender.com).
