# Supplementary figures and images for: Plant immunity to insect herbivores: mechanisms, interactions, and innovations for sustainable pest management
Source: Front Plant Sci. 2025 Jul 22;16:1599450. doi: 10.3389/fpls.2025.1599450 (PMC12321809; doi:10.3389/fpls.2025.1599450)

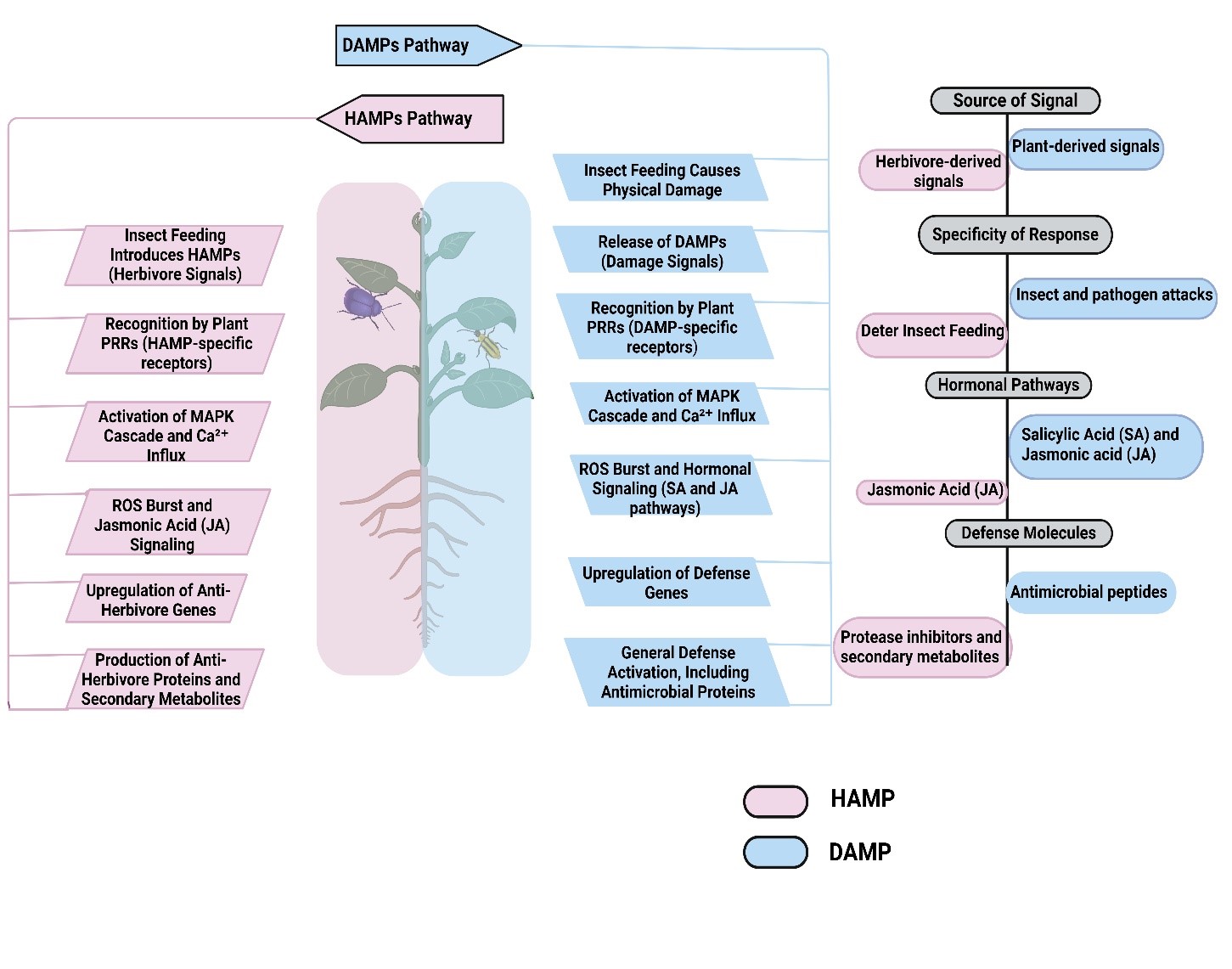

Supplement: Supplementary Figure 1 — Differentiation between damage-associated molecular pattern (DAMP) and herbivore-associated molecular pattern (HAMP) pathways during plant defense against insect attack. The chart illustrates the distinct pathways and mechanisms of DAMPs and HAMPs in triggering plant defense responses upon insect feeding (created using BioRender.com). [file Image1.jpeg]
